# International Union of Immunological Societies: 2017 Primary Immunodeficiency Diseases Committee Report on Inborn Errors of Immunity

**DOI:** 10.1007/s10875-017-0464-9

**Published:** 2017-12-11

**Authors:** Capucine Picard, H. Bobby Gaspar, Waleed Al-Herz, Aziz Bousfiha, Jean-Laurent Casanova, Talal Chatila, Yanick J. Crow, Charlotte Cunningham-Rundles, Amos Etzioni, Jose Luis Franco, Steven M. Holland, Christoph Klein, Tomohiro Morio, Hans D. Ochs, Eric Oksenhendler, Jennifer Puck, Mimi L. K. Tang, Stuart G. Tangye, Troy R. Torgerson, Kathleen E. Sullivan

**Affiliations:** 1 Center for the Study of Immunodeficiencies, Necker Hospital for Sick Children, Assistance Publique-Hôpitaux de Paris (APHP), Paris, France; 2 Laboratory of Lymphocyte Activation and Susceptibility to EBV, INSERM UMR1163, Imagine Institute, Necker Hospital for Sick Children, Paris Descartes University, Paris, France; 3 UCL Great Ormond Street Institute of Child Health, London, UK; 4 Department of Pediatrics, Faculty of Medicine, Kuwait University, Kuwait City, Kuwait; 5 Laboratoire d’Immunologie Clinique, d’Inflammation et d’Allergy LICIA Clinical Immunology Unit, Casablanca Children’s Hospital, Ibn Rochd Medical School, King Hassan II University, Casablanca, Morocco; 6 St. Giles Laboratory of Human Genetics of Infectious Diseases, Rockefeller Branch, The Rockefeller University, New York, NY USA; 7 Howard Hughes Medical Institute, New York, NY USA; 8 Laboratory of Human Genetics of Infectious Diseases, Necker Branch, INSERM UMR1163, Imagine Institute, Necker Hospital for Sick Children, University Paris Descartes, Paris, France; 9 Pediatric Hematology-Immunology Unit, Necker Hospital for Sick Children APHP, Paris, France; 10 Division of Immunology, Children’s Hospital Boston, Boston, MA USA; 11 Laboratory of Neuroinflammation and Neurogenetics, Necker Branch, INSERM UMR1163, Paris Descartes University, Sorbonne-Paris-Cité, Institut Imagine, Paris, France; 12 Division of Evolution and Genomic Sciences, School of Biological Sciences, Faculty of Biology, Medicine and Health, University of Manchester, Manchester Academic Health Science Centre, Manchester, UK; 13 Departments of Medicine and Pediatrics, Mount Sinai School of Medicine, NewYork, NY USA; 14 Ruth’s Children’s Hospital-Technion, Haifa, Israel; 15 Grupo de Inmunodeficiencias Primarias, Facultad de Medicina, Universidad de Antioquia UdeA, Medellin, Colombia; 16 Laboratory of Clinical Infectious Diseases, National Institute of Allergy and Infectious Diseases, Bethesda, MD USA; 17 Dr von Hauner Children’s Hospital, Ludwig-Maximilians-University Munich, Munich, Germany; 18 Department of Pediatrics and Developmental Biology, Tokyo Medical and Dental University (TMDU), Tokyo, Japan; 19 Department of Pediatrics, University of Washington and Seattle Children’s Research Institute, Seattle, WA USA; 20 Department of Clinical Immunology, Hôpital Saint-Louis, Assistance Publique-Hôpitaux de Paris, University Paris Diderot, Sorbonne Paris Cité, Paris, France; 21 Department of Pediatrics, University of California San Francisco and UCSF Benioff Children’s Hospital, San Francisco, CA USA; 22 Murdoch Children’s Research Institute, Melbourne, VIC Australia; 23 Department of Paediatrics, University of Melbourne, Melbourne, VIC Australia; 24 Department of Allergy and Immunology, Royal Children’s Hospital, Melbourne, Australia; 25 Immunology Division, Garvan Institute of Medical Research, Darlinghurst, NSW Australia; 26 St Vincent’s Clinical School, University of NSW, Sydney, Australia; 27 Division of Allergy Immunology, Department of Pediatrics, The Children’s Hospital of Philadelphia, University of Pennsylvania Perelman School of Medicine, ARC 1216-I 3615 Civic Center Blvd, Philadelphia, PA 19104 USA

**Keywords:** IUIS, primary immune deficiency, immune dysregulation, autoinflammatory disorders

## Abstract

Beginning in 1970, a committee was constituted under the auspices of the World Health Organization (WHO) to catalog primary immunodeficiencies. Twenty years later, the International Union of Immunological Societies (IUIS) took the remit of this committee. The current report details the categorization and listing of 354 (as of February 2017) inborn errors of immunity. The growth and increasing complexity of the field have been impressive, encompassing an increasing variety of conditions, and the classification described here will serve as a critical reference for immunologists and researchers worldwide.

## Introduction

**Fig. 1:**
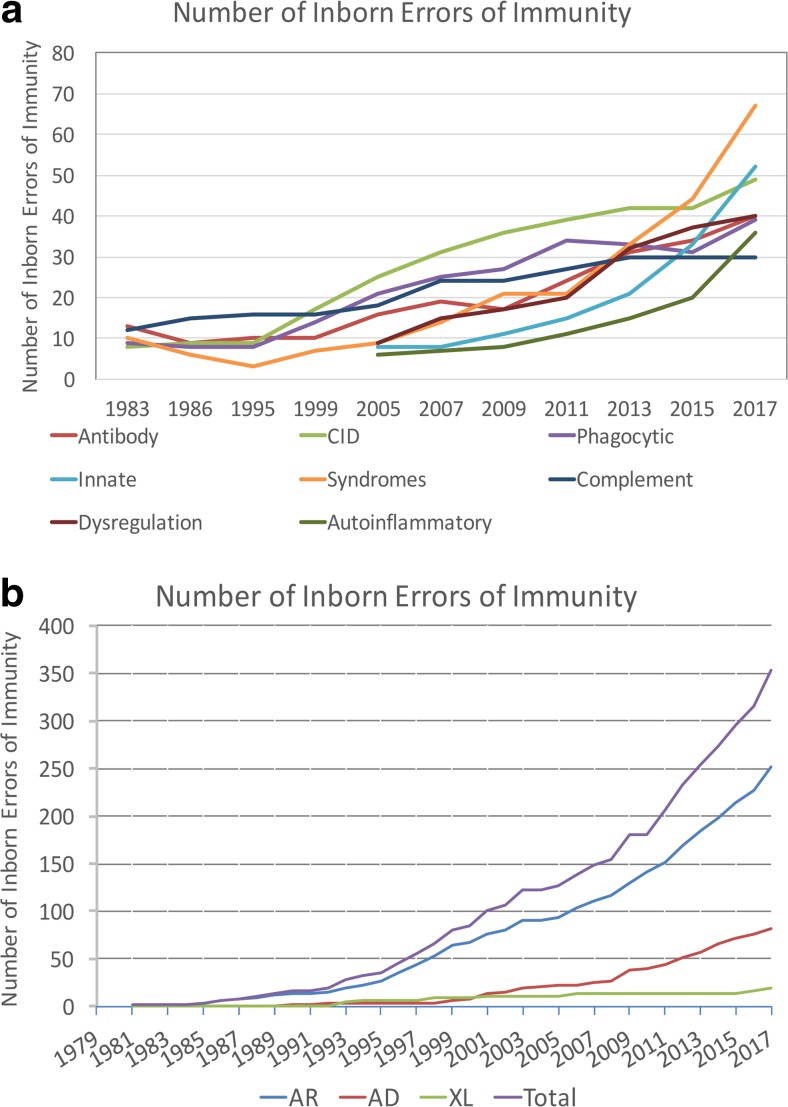
Each publication of the World Health Organization and IUIS Primary Immunodeficiencies Committee was reviewed for the number of conditions listed and displayed graphically [1–19]. The rapid increase in the twenty-first century relates to improved awareness and increasing use of sequencing. Assuming 20,000 coding genes in the human genome, inborn errors of immunity are implicated through mutations in 1.7% of these genes. There are now 330 specific disorders, 320 monogenic defects, 312 distinct genes (nine genes with both LOF and GOF and C4 deficiency requiring defects in both C4A and C4B). **a** The categorization of the inborn errors of immunity according the schema in the current manuscript. **b** The categorization of the inborn errors of immunity according to their inheritance

In 1970, Drs. Fudenberg, Good, Hitzig, Kunkel, Roitt, Rosen, Rowe, Seligmann, and Soothill met under the auspices of the World Health Organization to classify the emerging “primary immune deficiencies.” This august group focused on understanding whether immunodeficiencies could be categorized as B cell disorders or T cell disorders [1, 2]. Their initial report identified 16 distinct immunodeficiencies and included the prophetic comment that “the variable immunodeficiency group probably lumps together a series of syndromes…. Included in this group are cases previously classified as ‘congenital’, non-sex linked or sporadic hypogammaglobulinemia, primary ‘dysgammglobulinemia’ of both childhood and adult life, and ‘acquired’ primary hypogammaglobulinemia. It is hoped that careful analysis of such patients…. will result in delineation of several homogeneous syndromes…”. Indeed, the emergence of monogenic causes of hypogammaglobulinemia (Table 3) and disorders with variable immunoglobulin abnormalities associated with immune dysregulation (Table 4) have been the groups of immunodeficiencies most transformed by the advent of new technologies. Another group dramatically impacted by resetting of the clinical radar and new techniques has been the set of disorders associated with a limited spectrum of infectious susceptibility. The graphs in Fig. 1 define the transformation of the field over the interval during which next-generation sequencing came to prominence. The tremendous progress, energy, and enthusiasm in the field currently have led to a greater need than ever for a current cataloging of the disorders.

The new disorders (since 2015 [3]) represent an impressive spectrum of phenotypes. There are 354 distinct disorders with 344 different gene defects listed. The emerging dominance of next-generation sequencing has driven the rapid increase in the number of recognized disorders which has led to two major consequences. Often new inborn errors of immunity are initially described in a single kindred or a small number of kindreds. This may lead to incorrect assumptions about prevalence and phenotype. In fact, for most disorders, we have little idea of the prevalence within even the recognized population with the described phenotype. The second consequence of the rapid rise of next-generation sequencing is a striking expansion of the phenotypic spectrum associated with many diseases. Where once the phenotype of a given disorder was clear, the spectrum of manifestations often extends impressively once the ascertainment is not linked to a preconceived idea [20]. As a community, we recognize the importance of publishing cases and small series and to report specific mutations with clinical findings because publications are used to define likelihood of causality during bioinformatic analysis of next-generation sequencing results.

In 1999, the Committee on Primary Immunodeficiencies came under the auspices of the International Union of Immunological Societies (IUIS). The current committee met on February 23–24, 2017, in London to update the classification of human primary immunodeficiencies. Inclusion in this “master list” requires a body of literature supporting causality of a gene defect and a penetrance indicating clinical relevance [21]. Committee members vote on inclusion of each new disorder and this publications lists those included as of the February 2017 meeting. The landscape is changing so rapidly, and the number of primary immunodeficiencies growing so fast, that two major changes have been implemented. The published list will continue to serve as a reference; however, this list will now be available as a csv file on the IUIS website to enable sorting according to gene, disease name, or clinical/laboratory feature. This file will also include the associated ICD10 codes in order to promote harmonization of utilization. The second major change is to the nomenclature. The term primary immunodeficiency has an important legacy—the abbreviations PID or PIDD are often used by patient organizations and are recognized around the world. However, this terminology does limit the conceptualization of disorders to those in which susceptibility to infection is the main manifestation. The improving recognition of *immune dysregulation* diseases, including the growing field of autoinflammatory disorders and interferonopathies, has mandated that a more encompassing terminology be used. This manuscript, therefore, utilizes “inborn errors of immunity” as the descriptor for the work and the categorization. In addition to embracing technology to remain updated, the companion publication “Update of the Phenotypical IUIS Classification for Primary Immunodeficiencies” will provide a phenotype-oriented approach to the IUIS categorization of disorders. Moreover, a new free application can be found as “PID phenotypical diagnosis” or “PID classification” from iTunes and Android app stores [22, 23]. Information that is readily accessible is the new standard, and the IUIS Expert Committee on Primary Immunodeficiencies believes that improved access to information will positively impact patient care around the world.

The tables divide disease categories according to common phenotypes for ease of review and searching. Table 1 lists combined immunodeficiencies, Table 2 lists combined immunodeficiencies with syndromic features, Table 3 lists predominantly antibody deficiencies, Table 4 lists diseases of immune dysregulation, Table 5 lists defects of phagocyte number or function, Table 6 lists defects in intrinsic and innate immunity, Table 7 lists autoinflammatory diseases, Table 8 lists complement deficiencies, and Table 9 lists phenocopies of inborn errors of immunity. The division into phenotypes for the purpose of this list does not imply that the presentation is homogeneous. Each disorder is listed only once for the sake of simplicity although distinct modes of inheritance can be listed separately. There are nine genes for which both loss-of-function and gain-of-function variants have been identified: *CFB*, *C3*, *CARD11*, *STAT1*, *STAT3*, *WAS*, *JAK1*, *IFIH1*, and *ZAP70*. For these, the loss-of-function and gain-of-function aspects are listed. Within each table, there are additional sub-tables that segregate into coherent phenotypic sets. At the end of each table, the new disorders, added for this publication, are listed for easy reference. Other features important for navigation of the list include the use of OMIM links [24]. For additional information on a gene, the links can be accessed from within the online publication. For the second time, we also include non-inborn errors of immunity in Table 9, representing phenocopies of inborn errors which might be important to consider diagnostically.

**Table 1:** Immunodeficiencies affecting cellular and humoral immunity

Disease	Genetic defect	Inheritance	OMIM	T cells	B cells	Ig	Associated features
1. T-B+ severe combined immune deficiency (SCID)							
γc deficiency (common gamma chain SCID, CD132 deficiency)	*IL2RG*	XL	308380	Very low	Normal to high	Low	Low NK
JAK3 deficiency	*JAK3*	AR	600173	Very low	Normal to high	Low	Low NK
IL7Rα deficiency	*IL7R*	AR	146661	Very low	Normal to high	Low	Nl NK
CD45 deficiency	*PTPRC*	AR	151460	Very low	Normal	Low	Nl γ/δ Τ cells
CD3δ deficiency	*CD3D*	AR	186790	Very low	Normal	Low	Nl NK, no γ/δ T cells
CD3ε deficiency	*CD3E*	AR	186830	Very low	Normal	Low	Nl NK, no γ/δ T cells
CD3ζ deficiency	*CD247*	AR	186780	Very low	Normal	Low	Nl NK, no γ/δ T cells
Coronin-1A deficiency	*CORO1A*	AR	605000	Very low	Normal	Low	Detectable thymus, EBV
LAT deficiency	*LAT*	AR	602354	Nl to low number	Nl to low	High	Adenopathy, splenomegaly, recurrent infections, autoimmunity
2. T-B- SCID							
RAG1 deficiency	*RAG1*	AR	179615	Very low	Very low	Decreased	Nl NK
RAG2 deficiency	*RAG2*	AR	179616	Very low	Very low	Decreased	Nl NK
DCLRE1C (Artemis) deficiency	*DCLRE1C*	AR	605988	Very low	Very low	Decreased	Nl NK, radiation sensitive
DNA PKcs deficiency	*PRKDC*	AR	176977	Very low	Very low	Variable	Nl NK, radiation sensitive, microcephaly
Cernunnos/XLF deficiency	*NHEJ1*	AR	611290	Very low	Very low	Decreased	Nl NK, radiation sensitive, microcephaly
DNA ligase IV deficiency	*LIG4*	AR	601837	Very low	Very low	Decreased	Nl NK, radiation sensitive, microcephaly
Reticular dysgenesis	*AK2*	AR	103020	Very low	Nl to low	Decreased	Granulocytopenia and deafness
Adenosine deaminase (ADA) deficiency	*ADA*	AR	608958	Very low	Low, decreasing	Low, decreasing	Low NK, bone defects, may have pulmonary alveolar proteinosis, cognitive defects
3. Combined immunodeficiencies generally less profound than severe combined immunodeficiency							
DOCK2 deficiency	*DOCK2*	AR	603122	Low	Normal	IgG Nl or low, poor antibody responses	Nl NK cells, but defective function. Poor interferon responses in hematopoietic and non-hematopoietic cells
CD40 ligand deficiency (CD154)	*CD40LG* (*TNFSF5*)	XL	300386	Nl to low	sIgM^+^, IgD^+^ cells present, absent sIgG^+^, IgA^+^, and IgE^+^ cells	IgM normal or high, other Ig isotypes low	Neutropenia, thrombocytopenia, hemolytic anemia, opportunistic infections, biliary tract and liver disease, *Cryptosporidium* infections
CD40 deficiency	*CD40* (*TNFRSF5*)	AR	109535	Normal	sIgM^+^, IgD^+^ cells present, absent sIgG^+^, IgA^+^ and IgE^+^ cells	IgM normal or high, other Ig isotypes low	Neutropenia, opportunistic infections, gastrointestinal and biliary tract and liver disease, *Cryptosporidium* infections
ICOS deficiency	*ICOS*	AR	604558	Normal	Normal	Low	Recurrent infections, autoimmunity, gastroenteritis, granulomas
CD3γ deficiency	*CD3G*	AR	186740	Nl number, but low TCR expression	Normal	Normal	
CD8 deficiency	*CD8A*	AR	186910	Absent CD8, nl CD4	Normal	Normal	Recurrent infections, may be asymptomatic
ZAP-70 deficiency (ZAP70 LOF)	*ZAP70*	AR	176947	Low CD8, Nl CD4 number but poor function	Normal	Normal	May have immune dysregulation, autoimmunity
MHC class I deficiency	*TAP1*	AR	170260	Low CD8, Nl CD4, absent MHC I on lymphocytes	Normal	Normal	Vasculitis, pyoderma gangrenosum
MHC class I deficiency	*TAP2*	AR	170261	Low CD8, Nl CD4, absent MHC I on lymphocytes	Normal	Normal	Vasculitis, pyoderma gangrenosum
MHC class I deficiency	*TAPBP*	AR	601962	Low CD8, Nl CD4, absent MHC I on lymphocytes	Normal	Normal	Vasculitis, pyoderma gangrenosum
MHC class I deficiency	*B2M*	AR	109700	Low CD8, Nl CD4, absent MHC I on lymphocytes	Normal	Normal	Sinopulmonary infections, cutaneous granulomas. Absent β2m associated proteins MHC I, CD1a, CD1b, CD1c
MHC class II deficiency group A	*CIITA*	AR	600005	Low CD4 cells Absent MHC II expression on lymphocytes	Normal	Nl to low	Respiratory and gastrointestinal infections, liver/biliary tract disease
MHC class II deficiency group B	*RFXANK*	AR	603200	Low CD4 cells Absent MHC II expression on lymphocytes	Normal	Nl to low	Respiratory and gastrointestinal infections, liver/biliary tract disease
MHC class II deficiency group C	*RFX5*	AR	601863	Low CD4 cells Absent MHC II expression on lymphocytes	Normal	Nl to low	Respiratory and gastrointestinal infections, liver/biliary tract disease
MHC class II deficiency group D	*RFXAP*	AR	601861	Low CD4 cells Absent MHC II expression on lymphocytes	Normal	Nl to low	Respiratory and gastrointestinal infections, liver/biliary tract disease
DOCK8 deficiency	*DOCK8*	AR	243700	Low, poor proliferation, few, poorly functioning Treg	Low, low CD27+ memory B cells Poor peripheral B cell tolerance	Low IgM, Nl to high IgG and IgA, high IgE	Low NK cells with poor function, eosinophilia, recurrent infections, cutaneous viral, fungal and staphylococcal infections, severe atopy, cancer diathesis
Rhoh deficiency	*RHOH*	AR	602037	Nl number, low naïve T cells, restricted repertoire, poor proliferation to CD3	Normal	Normal	HPV infection, lung granulomas, molluscum contagiosum, lymphoma
MST1 deficiency	*STK4*	AR	614868	Low, low terminal differentiated effector memory (TEMRA) cells, low naïve T cells, poor proliferation	Low	High	Intermittent neutropenia, bacterial, viral (HPV), candidal infections, EBV lymphoproliferation, autoimmune cytopenias, lymphoma, congenital heart disease
TCRα deficiency	*TRAC*	AR	615387	Absent TCRαβ, all T cells are γδ, poor proliferation	Normal	Normal	Recurrent viral, bacterial, fungal infections, immune dysregulation and autoimmunity, diarrhea
LCK deficiency	*LCK*	AR	615758	Low CD4^+^, low Treg, restricted T cell repertoire, poor TCR signaling	Normal	Nl IgG and IgA, high IgM	Recurrent infections, immune dysregulation, autoimmunity
MALT1 deficiency	*MALT1*	AR	615468	Nl number, poor proliferation	Normal	Nl levels, poor specific antibody response	Bacterial, fungal and viral infections
CARD11 deficiency (LOF)	*CARD11*	AR	615206	Nl number, predominant naïve T cells, poor proliferation	Normal, transitional B cell predominance	Absent/low	*Pneumocystis jirovecii* pneumonia, bacterial and viral infections
BCL10 deficiency	*BCL10*	AR	616098	Nl number, low memory T and Treg cells, poor antigen and anti-CD3 proliferation	Nl number, decreased memory and switched B cells	Low	Recurrent bacterial and viral infections, candidiasis, gastroenteritis
BCL11B deficiency	*BCL11B*	AD	617237	Low, poor proliferation	Normal	Normal	Congenital abnormalities, neonatal teeth, dysmorphic facies, absent corpus callosum, neurocognitive deficits
IL-21 deficiency	*IL21*	AR	615767	Nl number, nl/low function	Low	Low IgG	Severe early-onset colitis, recurrent sinopulmonary infections
IL-21R deficiency	*IL21R*	AR	615207	Nl number, low cytokine production, poor antigen proliferation	Normal	Nl number, poor specific antibody responses	Recurrent infections, *Pneumocystis jiroveci*, *Cryptosporidium* infections and liver disease
OX40 deficiency	*TNFRSF4*	AR	615593	Nl numbers, low antigen specific memory CD4+	Nl numbers, low memory B cells	Normal	Impaired immunity to HHV8, Kaposi’s sarcoma
IKBKB deficiency	*IKBKB*	AR	615592	Nl number, absent Treg and γ/δ T cells, impaired TCR activation	Nl number, poor function	Low	Recurrent bacterial, viral, fungal infections, opportunistic infections
NIK deficiency	*MAP3K14*	AR	604655	Nl number, poor proliferation to antigen	Low, low switched memory B cells	Low Ig’s	Low NK number and function, recurrent bacterial, viral and *Cryptosporidium* infections
RelB deficiency	*RELB*	AR	604758	Nl number, poor diversity, poor function			Recurrent infections
Moesin deficiency	*MSN*	XL	300988	Nl number, defective migration, proliferation	Low number	Low Ig’s over time	Recurrent infections with bacteria, varicella, neutropenia
TFRC deficiency	*TFRC*	AR	616740	Nl number, poor proliferation	Nl number, low memory B cells	Low	Recurrent infections, neutropenia, thrombocytopenia

SCID/CID spectrum: Infants with SCID who have maternal T cell engraftment may have T cells in normal numbers that do not function normally; these cells may cause autoimmune cytopenias or graft versus host disease. Hypomorphic mutations in several of the genes that cause SCID may result in Omenn syndrome (OS), or “leaky” SCID, or still less profound combined immunodeficiency (CID) phenotypes. Both OS and leaky SCID can be associated with > 300 autologous T cells/μL of peripheral blood and reduced, rather than absent, proliferative responses when compared with typical SCID caused by null mutations. A spectrum of clinical findings including typical SCID, OS, leaky SCID, CID, granulomas with T lymphopenia, autoimmunity and CD4 T lymphopenia can be found in an allelic series of *RAG1* and other SCID-associated genes. Total number of disorders in Table 1: 49 (17 SCID, 32 CID). New disorders: 5, *MOESIN*, *BCL11B*, *TFRC*, *RELB*, *LAT*. Removed gene: UNC119 deficiency has been removed. The *UNC119* variant reported previously is a benign polymorphism in unaffected individuals

*SCID* severe combined immunodeficiency, *EBV* Epstein-Barr virus, *MHC* major histocompatibility complex, *HPV* human papillomavirus, *Treg* T regulatory cell, *Nl* normal, *XL* X-linked inheritance, *AR* autosomal recessive inheritance, *AD* autosomal dominant inheritance, *LOF* loss-of-function

**Table 2:** Combined immunodeficiencies with associated or syndromic features

Disease	Genetic defect	Inheritance	OMIM	T cells	B cells	Ig	Associated features
1. Immunodeficiency with congenital thrombocytopenia							
Wiskott-Aldrich syndrome (WAS LOF)	*WAS*	XL	300392	Progressive decrease in numbers, abnormal lymphocyte responses to anti-CD3	Normal numbers	Low IgM and antibody responses to polysaccharides, often high IgA and IgE	Thrombocytopenia with small platelets, recurrent bacterial and viral infections, bloody diarrhea, eczema, lymphoma, autoimmune disease, IgA nephropathy, vasculitis. XL thrombocytopenia is a mild form of WAS, and XL neutropenia is caused by missense mutations in the GTPase binding domain of WASp
WIP deficiency	*WIPF1*	AR	602357	Reduced, defective lymphocyte responses to anti-CD3	Normal or low	Normal, except for high IgE	Thrombocytopenia with or without small platelets, recurrent bacterial and viral infections, eczema, bloody diarrhea, WAS protein absent
ARPC1B deficiency	*ARPC1B*	AR	604223	Normal	Normal numbers	Normal except for high IgA and IgE	Mild thrombocytopenia with normal sized platelets, recurrent invasive infections, colitis, vasculitis, autoantibodies (ANA, ANCA), eosinophilia, defective Arp2/3, filament branching
2. DNA repair defects other than those listed in Table 1							
Ataxia-telangiectasia	*ATM*	AR	607585	Progressive decrease, abnormal proliferation to mitogens	Normal	Often low IgA, IgE and IgG subclasses, increased IgM monomers, antibodies variably decreased	Ataxia, telangiectasia, pulmonary infections, lymphoreticular and other malignancies, increased alpha fetoprotein, increased radiosensitivity, chromosomal instability and chromosomal translocations
Nijmegen breakage syndrome	*NBS1*	AR	602667	Progressive decrease	Variably reduced	Often low IgA, IgE, and IgG subclasses, increased IgM, antibodies variably decreased	Microcephaly, dysmorphic facies, lymphomas, solid tumors, increased radiosensitivity, chromosomal instability
Bloom Syndrome	*BLM* (*RECQL3*)	AR	604610	Normal	Normal	Low	Short stature, dysmorphic facies, sun-sensitive erythema, marrow failure, leukemia, lymphoma, chromosomal instability
Immunodeficiency with centromeric instability and facial anomalies, ICF1	*DNMT3B*	AR	602900	Decreased or normal, responses to PHA may be decreased	Decreased or normal	Hypogammaglobulinemia or agammaglobulinemia, variable antibody deficiency	
Immunodeficiency with centromeric instability and facial anomalies, ICF2	*ZBTB24*	AR	614064	Decreased or normal,	Decreased or normal	Hypogammaglobulinemia or agammaglobulinemia, variable antibody deficiency	
Immunodeficiency with centromeric instability and facial anomalies, ICF3	*CDCA7*	AR	609937	responses to PHA may be decreased	Decreased or normal	Hypogammaglobulinemia or agammaglobulinemia, variable antibody deficiency	
Immunodeficiency with centromeric instability and facial anomalies, ICF4	*HELLS*	AR	603946	Decreased or normal	Decreased or normal	Hypogammaglobulinemia or agammaglobulinemia, variable antibody deficiency	
PMS2 deficiency	*PMS2*	AR	600259	Normal	Low B cells, switched and non-switched	Low IgG and IgA, high IgM, abnormal antibody responses	Recurrent infections, café-au-lait spots, lymphoma, colorectal carcinoma, brain tumors
RNF168 deficiency (radiosensitivity, immune deficiency, dysmorphic features, learning difficulties [RIDDLE] syndrome)	*RNF168*	AR	612688	Normal	Normal	Low IgG or IgA	Short stature, mild defect of motor control to ataxia, normal intelligence to learning difficulties, mild facial dysmorphism to microcephaly, increased radiosensitivity
MCM4 deficiency	*MCM4*	AR	602638	Normal	Normal	Normal	NK cells: low number and function. Viral infections (EBV, HSV, VZV), short stature, B cell lymphoma, adrenal failure
POLE1 (polymerase ε subunit 1) deficiency (FILS syndrome)	*POLE*	AR	174762	Decreased T cell proliferation	Low memory B cells	Low IgG2 and IgM, lack of antibody to PPS	Recurrent respiratory infections, meningitis, facial dysmorphism, livido, short stature
POLE2 (polymerase ε subunit 2) deficiency	*POLE2*	AR	602670	Lymphopenia, lack of TRECS, absent proliferation in response to antigens	Very low	Hypogammaglobulinemia	Recurrent infections, disseminated BCG infections, autoimmunity (type 1 diabetes, hypothyroidism, facial dysmorphism
Ligase I deficiency	*LIG1*	AR	126391	Lymphopenia, decreased mitogen response	Normal	Low IgA and IgG Reduced antibody responses	Recurrent respiratory infections, growth retardation, sun sensitivity, lymphoma, radiation sensitivity
NSMCE3 deficiency	*NSMCE3*	AR	608243	Number decreased, poor response to mitogens and antigens	Normal	Normal Decreased Ab responses to PPS normal IgG, IgA, elevated IgM	Severe lung disease (possibly viral), thymic hypoplasia, chromosomal breakage, radiation sensitivity
ERCC6L2 (Hebo deficiency)	*ERCC6L2*	AR	615667	Lymphopenia	Low	Normal	Facial dysmorphism, microcephaly, bone marrow failure
GINS1 deficiency	*GINS1*	AR	610608	Low or normal	Low or normal	High IgA, low IgM and IgG	Neutropenia, IUGR, NK cells very low
3. Thymic defects with additional congenital anomalies							
DiGeorge/velocardiofacial syndrome Chromosome 22q11.2 deletion syndrome (22q11.2DS)	*Large deletion* (*3 Mb*) *typically in chromosome 22*	AD	602054	Decreased or normal, 5% have < 1500 CD3T cells/μL in neonatal period	Normal	Normal or decreased	Hypoparathyroidism, conotruncal cardiac malformation, velopalatal insufficiency, abnormal facies, intellectual disability
DiGeorge/velocardiofacial syndrome	Unknown	Sporadic		Decreased or normal	Normal	Normal or decreased	Hypoparathyroidism, conotruncal cardiac malformation, velopalatal insufficiency, abnormal facies, intellectual disability
TBX1 deficiency	*TBX1*	AD	602054	Decreased or normal	Normal	Normal or decreased	Hypoparathyroidism, conotruncal cardiac malformation, velopalatal insufficiency, abnormal facies, intellectual disability
CHARGE syndrome due to CHD7 deficiency	*CHD7*	AD	608892	Decreased or normal, response to PHA may be decreased	Normal	Normal or decreased	Coloboma, heart anomaly, choanal atresia, intellectual disability, genital and ear anomalies, CNS malformation, some are SCID-like and have low TRECs
CHARGE syndrome due to SEMA3E deficiency	*SEMA3E*	AD	608166	Decreased or normal, response to PHA may be decreased	Normal	Normal or decreased	Coloboma, heart anomaly, choanal atresia, intellectual retardation, genital and ear anomalies, CNS malformation, some are SCID-like and have low TRECs
CHARGE syndrome	Unknown			Decreased or normal, response to PHA may be decreased	Normal	Normal or decreased	Coloboma, heart anomaly, choanal atresia, intellectual disability, genital and ear anomalies, CNS malformation, some are SCID-like and have low TRECs
Winged helix nude FOXN1 deficiency	*FOXN1*	AR	600838	Very low	Normal	Decreased	Severe infections, abnormal thymic epithelium, immunodeficiency, congenital alopecia, nail dystrophy, neural tube defect
Chromosome 10p13-p14 deletion Syndrome (10p13-p14DS)	*Del10p13-p14*	AD	601362	Normal, rarely lymphopenia and decreased lymphoproliferation to mitogens and antigens, hypolastic thymus may be present	Normal	Normal	Hypoparathyroidism, renal disease, deafness, growth retardation, facial dysmorphism, cardiac defects may be present, recurrent infections +/−
4. Immuno-osseous dysplasias							
Cartilage hair hypoplasia (CHH)	*RMRP*	AR	157660	Varies from severely decreased (SCID) to normal, impaired lymphocyte proliferation	Normal	Normal or reduced, antibodies variably decreased	Short-limbed dwarfism with metaphyseal dysostosis, sparse hair, bone marrow failure, autoimmunity, susceptibility to lymphoma and other cancers, impaired spermatogenesis, neuronal dysplasia of the intestine
Schimke immuno-osseous dysplasia	*SMARCAL1*	AR	606622	Decreased	Normal	Normal	Short stature, spondiloepiphyseal dysplasia, intrauterine growth retardation, nephropathy, bacterial, viral, fungal infections, may present as SCID, bone marrow failure
MYSM1 deficiency	*MYSM1*	AR	612176	T cell lymphopenia, reduced naïve T cells	Immature B cells	Hypogammaglobulinemia	Short stature, recurrent infections, congenital bone marrow failure, myelodysplasia, immunodeficiency affecting B cells and granulocytes, skeletal anomalies, cataracts, developmental delay.
MOPD1 deficiency	*RNU4ATAC*	AR	601428	Normal	Normal	Normal, specific antibodies variably decreased	Recurrent bacterial infections, lymphadenopathy, spondyloepiphyseal dysplasia, extreme intrauterine growth retardation, retinal dystrophy, facial dysmorphism, may present with microcephaly
EXTL3 deficiency	*EXTL3*	AR		Reduced	Normal	Variably decreased	Platyspondyly, kyphosis, variable skeletal dysplasias, developmental delay
5. Hyper IgE syndromes (HIES)							
AD-HIES STAT3 deficiency (Job syndrome)	*STAT3*	AD LOF	102582	Normal overall, Th-17 and T-follicular helper cells decreased	Normal, reduced switched and non-switched memory B cells, BAFF expression increased	High IgE, specific antibody production decreased	Distinctive facial features (broad nasal bridge), bacterial infections (boils and pulmonary abscesses, pneumatoceles) due to *S. aureus*, pulmonary aspergillus, *Pneumocystis jirovecii*, eczema, mucocutaneous candidiasis, hyperextensible joints, osteoporosis and bone fractures, scoliosis, retention of primary teeth, coronary and cerebral aneurysm formation
Comel-Netherton syndrome	*SPINK5*	AR	605010	Normal	Low Switched and non-switched B cells	High IgE and IgA Antibody variably decreased	Congenital ichthyosis, bamboo hair, atopic diathesis, increased bacterial infections, failure to thrive
PGM3 deficiency	*PGM3*	AR	172100	CD8 and CD4 T cells may be decreased	Low B and memory B cells	Normal or elevated IgG and IgA, most high IgE, eosinophilia	Severe atopy, autoimmunity, bacterial and viral infections, skeletal anomalies dysplasia: short stature, brachydactyly, dysmorphic facial features, and intellectual disability cognitive impairment, hypomyelination
6. Dyskeratosis congenita (DKC), myelodysplasia, short telomeres							
XL-DKC due to dyskerin deficiency	*DKC1*	XL	300126	Progressive decrease	Progressive decrease	Variable hypogammaglobulinemia	Intrauterine growth retardation, microcephaly, nail dystrophy, sparse scalp hair and eyelashes, hyperpigmentation of skin, palmar hyperkeratosis, premalignant oral leukoplakia, pancytopenia, myelodysplasia, +/− recurrent infections. A severe phenotype with developmental delay and cerebellar hypoplasia known as Hoyeraal-Hreidarsson syndrome (HHS) may occur in some DKC patients
AR-DKC due to nucleolar protein family A member 2 (NHP2) deficiency	*NHP2*	AR	606470	Decreased	Variable	Variable	
AR-DKC due to nucleolar protein family A member 3 (NHP3) or NOP10 deficiency	*NOP10*	AR	606471	Decreased	Variable	Variable	
AD/AR-DKC due to regulator of telomere elongation (RTEL1) deficiency	*RTEL1*	AD or AR	608833	Decreased	Variable	Variable	
AD-DKC due to TERC deficiency	*TERC*	AD	602322	Variable	Variable	Variable	
AD/AR-DKC due to TERT deficiency	*TERT*	AD or AR	187270	Variable	Variable	Variable	
AD-DKC due to TINF2 deficiency	*TINF2*	AD	604319	Variable	Variable	Variable	
AD/AR-DKC due to TPP1 deficiency	*TPP1*	AD or AR	609377	Variable	Variable	Variable	
AR-DKC due to DCLRE1B deficiency	*DCLRE1B/SNM1/APOLLO:*	AR	609683	Variable	Variable	Variable	
AR-DKC due to PARN deficiency	*PARN*	AR (AD?)	604212	Variable	Variable	Variable	
AR-DKC due to WRAP53 deficiency	*WRAP53*	AR	612661	Not reported	Not reported	Not reported	
Coats plus syndrome due to STN1 deficiency	*STN1*	AR	613128	Variable	Variable	Not known	Intrauterine growth retardation, premature aging, pancytopenia, hypocellular bone marrow, gastrointestinal hemorrhage due to vascular ectasia, intracranial calcification, abnormal telomeres
Coats plus syndrome due to CTC1 deficiency	*CTC1*	AR	613129	Normal	Normal	Normal	Intrauterine growth retardation, sparse graying hair, dystrophic nails, trilinear bone marrow failure, osteopenia, gastrointestinal hemorrhage due to vascular ectasia, retinal telangiectasia, intracranial calcification, abnormal telomeres
SAMD9	*SAMD9*	AD (GOF)	617053	Not reported	Not reported	Not reported	IUGR with gonadal abnormalities, adrenal failure, MDS with chromosome 7 aberrations, predisposition to infections, enteropathy, absent spleen
SAMD9L	*SAMD9L*	AD (GOF)	159550	Normal	Low	Not reported	Cytopenia, predisposition to MDS with chromosome 7 aberrations, immunodeficiency, and progressive cerebellar dysfunction
7. Defects of vitamin B_12_ and folate metabolism							
Transcobalamin 2 deficiency	*TCN2*	AR	613441	Normal	Variable	Decreased	Megaloblastic anemia, pancytopenia, if untreated for prolonged periods results in intellectual disability
SLC46A1/PCFT deficiency causing hereditary folate malabsorption	*SLC46A1*	AR	229050	Variable numbers and activation profile	Variable	Decreased	Megaloblastic anemia, if untreated for prolonged periods results in intellectual disability
Methylene-tetrahydrofolate dehydrogenase 1 (MTHFD1) deficiency	*MTHFD1*	AR	172460	Low thymic output, normal in vitro proliferation	Low	Decreased/poor antibody responses to conjugated polysaccharide antigens	Recurrent bacterial infection, *Pneumocystis jirovecii*, megaloblastic anemia, neutropenia, seizures, intellectual disability, folate-responsive
8. Anhidrotic ectodermodysplasia with immunodeficiency (EDA-ID))							
EDA-ID due to NEMO /IKBKG deficiency (ectodermal dysplasia, immune deficiency)	*NEMO* (*IKBKG*)	XL	300248	Normal or decreased, TCR activation impaired	Normal Low memory and isotype switched B cells	Decreased, some with elevated IgA, IgM, poor specific antibody responses, absent antibody to polysaccharide antigens	Anhidrotic ectodermal dysplasia (in some), various infections (bacteria, mycobacteria, viruses and fungi), colitis, conical teeth, variable defects of skin, hair and teeth, monocyte dysfunction
EDA-ID due to IKBA GOF mutation	*IKBA* (*NFKBIA*)	AD GOF	164008	Normal total T cells, TCR activation impaired	Normal B cell numbers, impaired BCR activation, low memory and isotype switched B cells	Decreased IgG and IgA, elevated IgM, poor specific antibody responses, absent antibody to polysaccharide antigens	Anhidrotic ectodermal dysplasia, various infections (bacteria, mycobacteria, viruses and fungi), colitis, variable defects of skin, hair and teeth, T cell and monocyte dysfunction
9. Calcium channel defects							
ORAI-1 deficiency	*ORAI1*	AR	610277	Normal, defective TCR mediated activation	Normal	Normal	Autoimmunity, EDA, non-progressive myopathy
STIM1 deficiency	*STIM1*	AR	605921	Normal, defective TCR mediated activation	Normal	Normal	Autoimmunity, EDA, non-progressive myopathy
10. Other defects							
Purine nucleoside phosphorylase (PNP) deficiency	*PNP*	AR	164050	Progressive decrease	Normal	Normal or low	Autoimmune hemolytic anemia, neurological impairment
Immunodeficiency with multiple intestinal atresias	*TTC7A*	AR	609332	Variable, but sometimes absent low TRECs	Normal or low	Markedly decreased IgG, IgM, IgA	Bacterial (sepsis), fungal, viral infections, multiple intestinal atresias, often with intrauterine polyhydramnios and early demise, some with SCID phenotype
Hepatic veno-occlusive disease with immunodeficiency (VODI)	*SP110*	AR	604457	Normal (decreased memory T cells)	Normal (decreased memory B cells)	Decreased IgG, IgA, IgM, absent germinal centers and tissue plasma cells	Hepatic veno-occlusive disease, Susceptibility to *Pneumocystis jirovecii* pneumonia, CMV, candida, thrombocytopenia, hepatosplenomegaly, cerebrospinal leukodystrophy
Vici syndrome due to EPG5 deficiency	*EPG5*	AR	615068	Profound depletion of CD4+ cells	Defective	Decreased (particularly IgG2)	Agenesis of the corpus callosum, cataracts, cardiomyopathy, skin hypopigmentation, intellectual disability, microcephaly, recurrent infections, chronic mucocutaneous candidiasis
HOIL1 deficiency	*HOIL1* (*RBCK1*)	AR	610924	Normal numbers	Normal, decreased memory B cells	Poor antibody responses to polysaccharides	Bacterial infections, autoinflammation, amylopectinosis
HOIP deficiency	*RNF31*	AR	612487	Normal numbers	Normal, decreased memory B cells	decreased	Bacterial infections, autoinflammation, amylopectinosis, lymphangiectasia
Hennekam-lymphangiectasia-lymphedema syndrome due to CCBE1 deficiency	*CCBE1*	AR	612753	Low/variable	Low/variable	decreased	Lymphangiectasia and lymphedema with facial abnormalities and other dysmorphic features
Hennekam-lymphangiectasia-lymphedema syndrome due to FAT4 deficiency	*FAT4*	AR	612411	Low/variable	Low/variable	decreased	Lymphangiectasia and lymphedema with facial abnormalities and other dysmorphic features
STAT5b deficiency	*STAT5B*	AR	604260	Modestly decreased	Normal	Normal	Growth-hormone insensitive dwarfism, dysmorphic features, eczema, lymphocytic interstitial pneumonitis, autoimmunity
Kabuki syndrome 1 due to KMT2D deficiency	*KMT2D* (*MLL2*)	AD	602113	Normal	Normal	Low IgA and occasionally low IgG	Typical facial abnormalities, cleft or high arched palate, skeletal abnormalities, short stature, intellectual disability, congenital heart defects, recurrent infections (otitis media, pneumonia) in 50% of patients. Autoimmunity may be present
Kabuki syndrome 2 due to KDM6A deficiency	*KDM6A*	XL (females may be affected)	300128	Normal	Normal	Low IgA and occasionally IgG	

Pure bone marrow failure syndromes have not been included. Total number of disorders in Table 2: 67. New disorders: 23, *ARPC1B*, *CDCA7*, *HELLS*, *POLE2*, *LIG1*, *GINS1*, *NSMCE3*, *ERCC6L2*, *TBX1*, *MYSM1*, *MOPD1*, *STN1*, *CTC1*, *KMT2D*, *KDM6A*, *SAMD9*, *SAMD9L*, *EXTL3*, *WRAP53*, *FAT4*. Unknown cause of DiGeorge syndrome, unknown cause CHARGE, 10p13-14 deletion

*IUGR* intrauterine growth retardation, *HSV* herpes simplex virus, *VZV* varicella zoster virus, *BCG* Bacillus Calmette-Guerin, *XL* X-linked inheritance, *AR* autosomal recessive inheritance, *AD* autosomal dominant inheritance, *LOF* loss-of-function, *GOF* gain-of-function

**Table 3:** Predominantly antibody deficiencies

Disease	Genetic defect	Inheritance	OMIM	Ig	Associated features
1. Severe reduction in all serum immunoglobulin isotypes with profoundly decreased or absent B cells, agammaglobulinemia					
BTK deficiency, X-linked agammaglobulinemia (XLA)	*BTK*	XL	300300	All isotypes decreased in majority of patients, some patients have detectable immunoglobulins	Severe bacterial infections, normal numbers of pro-B cells
μ heavy chain deficiency	*IGHM*	AR	147020	All isotypes decreased	Severe bacterial infections, normal numbers of pro-B cells
λ5 deficiency	*IGLL1*	AR	146770	All isotypes decreased	Severe bacterial infections, normal numbers of pro-B cells
Igα deficiency	*CD79A*	AR	112205	All isotypes decreased	Severe bacterial infections, normal numbers of pro-B cells
Igβ deficiency	*CD79B*	AR	147245	All isotypes decreased	Severe bacterial infections, normal numbers of pro-B cells
BLNK deficiency	*BLNK*	AR	604515	All isotypes decreased	Severe bacterial infections, normal numbers of pro-B cells
PIK3R1 deficiency	*PIK3R1*	AR	171833	All isotypes decreased	Severe bacterial infections, decreased or absent pro-B cells
E47 transcription factor deficiency	*TCF3*	AD	147141	All isotypes decreased	Recurrent bacterial infections
2. Severe reduction in at least 2 serum immunoglobulin isotypes with normal or low number of B cells, CVID phenotype					
Common variable immune deficiency with no gene defect specified (CVID)	*Unknown*	Variable		Low IgG and IgA and/or IgM	Clinical phenotypes vary: most have recurrent infections, some have polyclonal lymphoproliferation, autoimmune cytopenias and/or granulomatous disease
PIK3CD mutation (GOF)	*PIK3CD GOF*	AD	602839	All isotypes decreased	Severe bacterial infections; decreased or absent pro-B cells, EBV
PIK3R1 deficiency (LOF)	*PIK3R1*	AD	616005	All isotypes decreased	Severe bacterial infections, pro-B cells present and low numbers of memory B cells, EBV
PTEN Deficiency (LOF)	*PTEN*	AD	601728	Decreased	Lymphoproliferation, autoimmunity
CD19 deficiency	*CD19*	AR	107265	Low IgG and IgA and/or IgM	Recurrent infections, may have glomerulonephritis
CD81 deficiency	*CD81*	AR	186845	Low IgG, low or normal IgA and IgM	Recurrent infections, may have glomerulonephritis
CD20 deficiency	*MS4A1*	AR	112210	Low IgG, normal or elevated IgM and IgA	Recurrent infections
CD21 deficiency	*CR2*	AR	120650	Low IgG, impaired anti-pneumococcal response	Recurrent infections
TACI deficiency	*TNFRSF13B* (*TACI*)	AD or AR	604907	Low IgG and IgA and/or IgM	Variable clinical expression
BAFF receptor deficiency	*TNFRSF13C* (*BAFF-R*)	AR	606269	Low IgG and IgM,	Variable clinical expression
TWEAK deficiency	*TNFSF12*	AD	602695	Low IgM and A, lack of anti-pneumococcal antibody	Pneumonia, bacterial infections, warts, thrombocytopenia. Neutropenia
Mannosyl-oligosaccharide glucosidase deficiency (MOGS)	*MOGS* (*GCS1*)	AR	601336	Severe hypogammaglobulinemia,	Bacterial and viral infections, severe neurologic disease, also known as congenital disorder of glycosylation type IIb (CDG-IIb)
TRNT1 deficiency	*TRNT1*	AR	612907	B cell deficiency and hypogammaglobulinemia	Congenital sideroblastic anemia, deafness, developmental delay
TTC37 deficiency	*TTC37*	AR	614649	Poor antibody response to pneumococcal vaccine	Recurrent bacterial and viral infections, abnormal hair findings: trichorrhexis nodosa
NFKB1 deficiency	*NFKB1*	AD	164011	Normal or low IgG, IgA, IgM, low or normal B cells, low memory B cells	Recurrent sinopulmonary infections, COPD, EBV proliferation, autoimmune cytopenias, alopecia and autoimmune thyroiditis
NFKB2 deficiency	*NFKB2*	AD	615577	Low serum IgG, A and M; low B cell numbers	Recurrent sinopulmonary infections, alopecia, and endorinopathies
IKAROS deficiency	*IKZF1*	AD	603023	Low IgG, IgA, IgM, low or normal B cells, potentially reducing levels with age	Recurrent sinopulmonary infections
IRF2BP2 deficiency	*IRF2BP2*	AD	615332	Hypogammaglobulenia, absent IgA	Recurrent infections, possible autoimmunity and inflammatory disease
ATP6AP1 deficiency	*ATP6AP1*	XL	300197	Variable immunoglobulin findings	Hepatopathy, leukopenia, low copper
3. Severe reduction in serum IgG and IgA with normal/elevated IgM and normal numbers of B cells, hyper IgM					
AID deficiency	*AICDA*	AR	605257	IgG and IgA decreased, IgM increased	Bacterial infections, enlarged lymph nodes and germinal centers
UNG deficiency	*UNG*	AR	191525	IgG and IgA decreased, IgM increased	Enlarged lymph nodes and germinal centers
INO80	*INO80*	AR	610169	IgG and IgA decreased, IgM increased	Severe bacterial infections
MSH6	*MSH6*	AR	600678	Variable IgG, defects, increased IgM in some, normal B cells, low switched memory B cells, Ig-class switch recombination and somatic hypermutation defects	Family or personal history of cancer
4. Isotype, light chain, or functional deficiencies with generally normal numbers of B cells					
Ig heavy chain mutations and deletions	*Mutation or chromosomal deletion at 14q32*	AR		One or more IgG and/or IgA subclasses as well as IgE may be absent	May be asymptomatic
Kappa chain deficiency	*IGKC*	AR	147200	All immunoglobulins have lambda light chain	Asymptomatic
Isolated IgG subclass deficiency	Unknown	?		Reduction in one or more IgG subclass	Usually asymptomatic, a minority may have poor antibody response to specific antigens and recurrent viral/bacterial infections
IgG subclass deficiency with IgA deficiency	Unknown	?		Reduced IgA with decrease in one or more IgG subclass	Recurrent bacterial infections
Selective IgA deficiency	Unknown	?		Very low to absent IgA with other isotypes normal, normal subclasses and specific antibodies	Bacterial infections, autoimmunity mildly increased
Specific antibody deficiency with normal Ig levels and normal B cells	Unknown	?		Normal	Reduced ability to produce antibodies to specific antigens
Transient hypogammaglobulinemia of infancy	Unknown	?		IgG and IgA decreased	Normal ability to produce antibodies to vaccine antigens, usually not associated with significant infections
CARD11 GOF	*CARD11*	AD GOF	607210	High B cell numbers due to constitutive NF-κB activation	Splenomegaly, lymphadenopathy, poor vaccine response
Selective IgM deficiency	Unknown	?		Absent serum IgM	Pneumococcal / bacterial infections

Common variable immunodeficiency disorders (CVID) include several clinical and laboratory phenotypes that may be caused by distinct genetic and/or environmental factors. Some patients with CVID and no known genetic defect have markedly reduced numbers of B cells as well as hypogammaglobulinemia. Identification of causal variants can assist in defining treatment. In addition to monogenic causes on this table, a small minority of patients with XLP (Table 4), WHIM syndrome (Table 6), ICF (Table 2), VOD1 (Table 2), thymoma with immunodeficiency (Good syndrome) or myelodysplasia are first seen by an immunologist because of recurrent infections, hypogammaglobulinemia and normal or reduced numbers of B cells. Total number of disorders in Table 3: 40. New disorders: 7, *PTEN*, *NFKB1*, *IKZF1*, *IRF2BP2*, *ATP6AP1.* Selective igA deficiency, selective IgM deficiency

*EBV* Epstein-Barr virus, *COPD* chronic obstructive pulmonary disease, *XL* X-linked inheritance, *AR* autosomal recessive inheritance, *AD* autosomal dominant inheritance, *LOF* loss-of-function, *GOF* gain-of-function

**Table 4:** Diseases of immune dysregulation

Disease	Genetic defect	Inheritance	OMIM	Circulating T cells	Circulating B cells	Functional defect	Associated features
1. Familial hemophagocytic lymphohistiocytosis (FHL syndromes)							
Perforin deficiency (FHL2)	*PRF1*	AR	170280	Increased activated T cells	Normal	Decreased to absent NK and CTL activities cytotoxicity	Fever, (H)SM, hemophagocytic lymphohistiocytosis (HLH), cytopenias
UNC13D/Munc13-4 deficiency (FHL3)	*UNC13D*	AR	608897	Increased activated T cells	Normal	Decreased to absent NK and CTL activities (cytotoxicity and/or degranulation)	Fever, (H)SM, HLH, cytopenias,
Syntaxin 11 deficiency (FHL4)	*STX11*	AR	605014	Increased activated T cells	Normal	Decreased NK activity (cytotoxicity and/or degranulation)	Fever, (H)SM, cHLH, cytopenias,
STXBP2/Munc18-2 deficiency (FHL5)	*STXBP2*	AR or AD	601717	Increased activated T cells	Normal	Decreased NK and CTL activities (cytotoxicity and/or degranulation)	Fever, (H)SM, cHLH, cytopenias, enteropathy
FAAP24 deficiency	*FAAP24*	AR	610884	Increased activated T cells	Normal	Failure to kill autologous EBV transformed B cells. Normal NK cell function	EBV infection-driven lymphoproliferative disease
2. FHL syndromes with hypopigmentation							
Chediak-Higashi syndrome	*LYST*	AR	606897	Increased activated T cells	Normal	Decreased NK and CTL activities (cytotoxicity and/or degranulation)	Partial albinism, recurrent infections, fever, HSM, HLH, giant lysosomes, neutropenia, cytopenias, bleeding tendency, progressive neurological dysfunction
Griscelli syndrome, type 2	*RAB27A*	AR	603868	Normal	Normal	Decreased NK and CTL activities (cytotoxicity and/or degranulation)	Partial albinism, fever, HSM, HLH, cytopenias
Hermansky-Pudlak syndrome, type 2	*AP3B1*	AR	603401	Normal	Normal	Decreased NK and CTL activities (cytotoxicity and/or degranulation)	Partial albinism, recurrent infections, pulmonary fibrosis, increased bleeding, neutropenia, HLH
Hermansky-Pudlak syndrome, type 10	*AP3D1*	AR	617050	Normal	Normal	Decreased NK and CTL activities (cytotoxicity and/or degranulation)	Oculocutaneous albinism, severe neutropenia, recurrent infections, seizures, hearing loss, and neurodevelopmental delay
3. Regulatory T cell defects							
IPEX, immune dysregulation, polyendocrinopathy, enteropathy X-linked	*FOXP3*	XL	300292	Normal	Normal	Lack of (and/or impaired function of) CD4^+^ CD25^+^ FOXP3^+^ regulatory T cells (Tregs)	Autoimmune enteropathy, early-onset diabetes, thyroiditis hemolytic anemia, thrombocytopenia, eczema, elevated IgE, IgA
CD25 deficiency	*IL2RA*	AR	147730	Normal to decreased	Normal	No CD4 + C25+ cells with impaired function of Tregs cells	Lymphoproliferation, autoimmunity, impaired T cell proliferation
CTLA4 deficiency (ALPSV)	*CTLA4*	AD	123890	Decreased	Decreased	Impaired function of Tregs.	Autoimmune cytopenias, enteropathy, interstitial lung disease, extra-lymphoid lymphocytic infiltration recurrent infections
LRBA deficiency	*LRBA*	AR	606453	Normal or decreased CD4 numbers, T cell dysregulation	Low or normal numbers of B cells	Reduced I IgG and IgA in most	Recurrent infections, inflammatory bowel disease, autoimmunity, EBV infections
STAT3 GOF mutation	*STAT3*	AD (GOF)	102582	Decreased	Decreased	Enhanced STAT3 signaling, leading to increased Th17 cell differentiation, lymphoproliferation and autoimmunity. Decreased Tregs and impaired function	Lymphoproliferation, solid organ autoimmunity, recurrent infections
BACH2 deficiency	*BACH2*	AD	605394	Progressive T cell lymphopenia	Impaired memory B cell development	Haplosufficiency for a critical lineage specification transcription factor	Lymphocytic colitis, sinopulmonary infections
4. Autoimmunity with or without Lymphoproliferation							
APECED (APS-1), autoimmune polyendocrinopathy with candidiasis and ectodermal dystrophy	*AIRE*	AR or AD	607358	Normal	Normal	AIRE serves as check-point in the thymus for negative selection of autoreactive T cells and for generation of Tregs	Autoimmunity: hypoparathyroidism hypothyroidism, adrenal insufficiency, diabetes, gonadal dysfunction and other endocrine abnormalities, chronic mucocutaneous candidiasis, dental enamel hypoplasia, alopecia areata enteropathy, pernicious anemia
ITCH deficiency	*ITCH*	AR	606409	Not assessed	Not assessed	Itch deficiency may cause immune dysregulation by affecting both anergy induction in autoreactive effector T cells and generation of Tregs	Early-onset chronic lung disease (interstitial pneumonitis), autoimmunity (thyroiditis, type I diabetes, chronic diarrhea/enteropathy, and hepatitis), failure to thrive, developmental delay, dysmorphic facial features
ZAP-70 combined hypomorphic and activation mutations	*ZAP70*	AR (LOF/GOF)	176947	Decreased CD8, normal or decreased CD4 cells	Normal or decreased	Hyperactive Zap70 kinase	Severe autoimmunity
Tripeptidyl-peptidase II deficiency	*TPP2*	AR	190470	Decreased	Decreased	TPP2 deficiency results in premature immunosenescence and immune dysregulation	Variable lymphoproliferation, severe autoimmune cytopenias, hypergammaglobulinemia, recurrent infections
JAK1 GOF	*JAK1*	AD GOF	147795	Not assessed	Not assessed	Hyperactive JAK1	HSM, eosinophilia, eosinophilic enteritis, thyroid disease, poor growth, viral infections
Prolidase deficiency	*PEPD*	AR	613230	Normal	Normal	Peptidase D	Autoantibodies common, chronic skin ulcers, eczema, infections
5. Autoimmune lymphoproliferative syndrome (ALPS, Canale-Smith syndrome)							
ALPS-FAS	*TNFRSF6*	AD or AR	134637	Increased CD4^−^CD8^−^TCR α/β-double negative (DN) T cells	Normal, low memory B cells	Apoptosis defect FAS mediated	Splenomegaly, adenopathies, autoimmune cytopenias, increased lymphoma risk, IgG and A normal or increased, elevated serum FasL and IL-10, vitamin B_12_
ALPS-FASLG	*FASLG*	AR	134638	Increased DN T cells	Normal	Apoptosis defect FAS mediated	Splenomegaly, adenopathies, autoimmune cytopenias, SLE, soluble FasL is not elevated
ALPS-caspase 10	*CASP10*	AD	601762	Increased DN T cells	Normal	Defective lymphocyte apoptosis	Adenopathies, splenomegaly, autoimmunity
ALPS-caspase 8	*CASP8*	AR	601763	Slightly increased DN T cells	Normal	Defective lymphocyte apoptosis and activation	Adenopathies, splenomegaly, bacterial and viral infections, hypogammaglobulinemia
FADD deficiency	*FADD*	AR	602457	Increased DN T cells	Normal	Defective lymphocyte apoptosis	Functional hyposplenism, bacterial and viral infections, recurrent episodes of encephalopathy and liver dysfunction
6. Immune dysregulation with colitis							
IL-10 deficiency	*IL10*	AR	124092	Normal	Normal	No functional IL-10 secretion	Inflammatory bowel disease (IBD), Folliculitis, recurrent respiratory diseases, arthritis,
IL-10Ra deficiency	*IL10RA*	AR	146933	Normal	Normal	Leukocytes unresponsive to IL-10	IBD, Folliculitis, recurrent respiratory diseases, arthritis, lymphoma
IL-10Rb deficiency	*IL10RB*	AR	123889	Normal	Normal	Leukocytes unresponsive to IL-10, IL-22, IL-26, IL-28A, IL-28B, and IL-29	IBD, folliculitis, recurrent respiratory diseases, arthritis, lymphoma
NFAT5 haploinsufficiency	*NFAT5*	AD	604708	Normal	Normal	Decreased memory B cells and plasmablasts	IBD, recurrent sinopulmonary infections
7. Susceptibility to EBV and lymphoproliferative conditions							
SH2D1A deficiency (XLP1)	*SH2D1A*	XL	300490	Normal or increased activated T cells	Reduced memory B cells	normal NK cell and CTL cytotoxic activity	Clinical and immunologic features triggered by EBV infection: HLH, lymphoproliferation, aplastic anemia, lymphoma. hypogammaglobulinemia, absent iNKT cells
XIAP deficiency (XLP2)	*XIAP*	XL	300079	Normal or Increased activated T cells; low/normal iNKT cells	Normal or reduced memory B cells	Increased T cells susceptibility to apoptosis to CD95 and enhanced activation-induced cell death (AICD)	EBV infection, splenomegaly, lymphoproliferation HLH, colitis, IBD, hepatitis low iNKT cells, hypogammaglobulinemia
CD27 deficiency	*CD27*	AR	615122	Normal	No memory B cells	Low immunoglobulin after EBV infection	Features triggered by EBV infection, HLH, aplastic anemia, low iNKT cells, lymphoma
CTPS1 deficiency	*CTPS1*	AR	615897	Nl to low, poor proliferation to antigen	Nl/low	Nl/high IgG	Recurrent/chronic bacterial and viral infections (EBV, VZV), lymphoproliferation, B cell non-Hodgkin lymphoma
RASGRP1 deficiency	*RASGRP1*	AR	603962	Poor activation, proliferation, motility	Poor activation, proliferation, motility	Normal IgM, IgG, increased IgA	Recurrent pneumonia, herpesvirus infections, EBV associated lymphoma
CD70 deficiency	*CD70* (*TNFSF7*)	AR	602840	Nl number, low Treg, poor activation and function	Nl number, poor antibody and memory responses	Reduced IgM, IgG, IgA (75%) and reduced Ag-specific Ab responses (50%)	EBV susceptibility, Hodgkin lymphoma
RLTPR (CARMIL2) deficiency	*RLTPR*	AR	610859	Nl number, low Treg, high CD4, poor function	Nl number	Nl to low, poor T dependent antibody response	Recurrent bacterial, fungal and mycobacterial infections, viral warts, molluscum and EBV lymphoproliferative and other malignancy, atopy
ITK deficiency	*ITK*	AR	186973	Progressive decrease	Normal	Nl to low	EBV associated B cell lymphoproliferation, lymphoma, Nl or low IgG
MAGT1 deficiency (XMEN)	*MAGT1*	XL	300853	Low CD4 Low recent thymic emigrant cells, poor proliferation to CD3	Normal	Normal	EBV infection, lymphoma, viral infections, respiratory and GI infections
PRKCD deficiency	*PRKCD*	AR	176977	Normal	Low memory B cells, high CD5 B cells	Apoptotic defect in B cells	Recurrent infections, EBV chronic infection, lymphoproliferation, SLE-like autoimmunity (nephrotic and antiphospholipid syndromes), low IgG

Total number of disorders in Table 4: 40. New disorders: 9, *FAAP24*, *RASGRP1*, *CD70*, *RLTPR*, *ZAP70* (GOF + LOF), *AP3D1*, *BACH2*, *JAK1 GOF*, *PEPD*. Removed gene: Hermansky-Pudlak syndrome type 9 was removed due to retraction of the defining publication

*FHL* familial hemophagocytic lymphohistiocytosis, *HLH* hemophagocytic lymphohistiocytosis, *HSM* hepatosplenomegaly ((H)SM indicating variable hepatomegaly), *DN* double negative, *SLE* systemic lupus erythematous, *IBD* inflammatory bowel disease, *XL* X-linked inheritance, *AR* autosomal recessive inheritance, *AD* autosomal dominant inheritance, *LOF* loss-of-function, *GOF* gain-of-function

**Table 5:** Congenital defects of phagocyte number or function

Disease	Genetic defect	Inheritance	OMIM	Affected cells	Affected function	Associated features
1. Congenital neutropenias						
Elastase deficiency (SCN1)	*ELANE*	AD	130130	N	Myeloid differentiation	Susceptibility to MDS/leukemia Severe congenital neutropenia or cyclic neutropenia
GFI 1 deficiency (SCN2)	*GFI1*	AD	600871	N	Myeloid differentiation	B/T lymphopenia
HAX1 deficiency (Kostmann disease) (SCN3)	*HAX1*	AR	605998	N	Myeloid differentiation	Cognitive and neurological defects in patients with defects in both HAX1 isoforms, susceptibility to MDS/leukemia
G6PC3 deficiency (SCN4)	*G6PC3*	AR	611045	N	Myeloid differentiation, chemotaxis, O_2_^−^ production	Structural heart defects, urogenital abnormalities, inner ear deafness, and venous angiectasias of trunks and limbs
VPS45 deficiency (SCN5)	*VPS45*	AR	610035	N	Myeloid differentiation, migration	Extramedullary hematopoiesis, bone marrow fibrosis, nephromegaly
Glycogen storage disease type 1b	*G6PT1*	AR	602671	N + M	Myeloid differentiation, chemotaxis, O_2_^−^ production	Fasting hypoglycemia, lactic acidosis, hyperlipidemia, hepatomegaly
X-linked neutropenia/myelodysplasia WAS GOF	*WAS*	XL	300392	N	Differentiation, mitosis	Neutropenia, myeloid maturation arrest, monocytopenia, variable lymphoid anomalies
P14/LAMTOR2 deficiency	*LAMTOR2*	AR	610389	N + M	Endosomal biogenesis	Neutropenia Hypogammaglobulinemia ↓CD8 cytotoxicity, partial albinism, growth failure
Barth syndrome (3-methylglutaconic aciduria type II)	*TAZ*	XL	300394	N + L Mel	Mitochondrial function	Cardiomyopathy, myopathy, growth retardation, neutropenia
Cohen syndrome	*VPS13B*	AR	607817	N	Myeloid differentiation	Dysmorphism, mental retardation, obesity, deafness, neutropenia
Clericuzio syndrome (poikiloderma with neutropenia)	*USB1*	AR	613276	N	Myeloid differentiation	Retinopathy, developmental delay, facial dysmorphisms, poikiloderma
JAGN1 deficiency	*JAGN1*	AR	616012	N	Myeloid differentiation	Myeloid maturation arrest, osteopenia
3-Methylglutaconic aciduria	*CLPB*	AR	616254	N	Myeloid differentiation Mitochondrial protein	Neurocognitive developmental aberrations, microcephaly, hypoglycemia, hypotonia, ataxia, seizures, cataracts, IUGR
G-CSF receptor deficiency	*CSF3R*	AR	138971	N	Stress granulopoiesis disturbed	
SMARCD2 deficiency	*SMARCD2*	AR	601736	N	Chromatin remodeling, myeloid differentiation and neutrophil functional defect	Neutropenia, developmental aberrations, skeletal abnormalities, hematopoietic stem cells, myelodysplasia
HYOU1 deficiency	*HYOU1*	AR	601746	N	Unfolded protein response	Hypoglycemia, inflammatory complications
2. Defects of motility						
Leukocyte adhesion deficiency type 1 (LAD1)	*ITGB2*	AR	600065	N + M +L + NK	Adherence, chemotaxis, endocytosis, T/NK cytotoxicity	Delayed cord separation, skin ulcers, periodontitis, leukocytosis
Leukocyte adhesion deficiency type 2 (LAD2)	*SLC35C1*	AR	605881	N + M	Rolling, chemotaxis	Mild LAD type 1 features with hh-blood group, growth retardation, developmental delay
Leukocyte adhesion deficiency type 3 (LAD3)	*FERMT3*	AR	607901	N + M + L + NK	Adherence, chemotaxis	LAD type 1 plus bleeding tendency
Rac 2 deficiency	*RAC2*	AD	602049	N	Adherence, chemotaxis O_2_^−^ production	Poor wound healing, leukocytosis
β actin deficiency	*ACTB*	AD	102630	N + M	Motility	Mental retardation, short stature
Localized juvenile periodontitis	*FPR1*	AR	136537	N	Formylpeptide induced chemotaxis	Periodontitis only
Papillon-Lefèvre syndrome	*CTSC*	AR	602365	N + M	Chemotaxis	Periodontitis, palmoplantar hyperkeratosis in some patients
Specific granule deficiency	*CEBPE*	AR	189965	N	Chemotaxis	Neutrophils with bilobed nuclei
Shwachman-Diamond syndrome	*SBDS*	AR	607444	N	Chemotaxis	Pancytopenia, exocrine pancreatic insufficiency, chondrodysplasia
WDR1 deficiency	*WDR1*	AR	604734	N	Spreading, survival, chemotaxis	Mild neutropenia, poor wound healing, severe stomatitis, neutrophil nuclei herniate
Cystic fibrosis	*CFTR*	AR	602421	M only	Chemotaxis	Respiratory infections, pancreatic insufficiency, elevated sweat chloride
Schwachman Diamond syndrome due to DNAJC21 deficiency	*DNAJC21*	AR	617048	N	Motility, ribosome biogenesis	Metaphyseal changes, short stature, developmental delay, pancreatic dysfunction, bone marrow failure
Neutropenia with combined immune deficiency due to MKL1 deficiency	*MKL1*	AR	606078	N + M +L + NK	Impaired expression of cytoskeletal genes	Mild thrombocytopenia
3. Defects of respiratory burst						
X-linked chronic granulomatous disease (CGD), gp91phox	*CYBB*	XL	300481	N + M	Killing (faulty O_2_^−^ production)	Infections, autoinflammatory phenotype, IBD McLeod phenotype in patients with deletions extending into the contiguous Kell locus
Autosomal recessive CGD p22phox	*CYBA*	AR	608508	N + M	Killing (faulty O_2_^−^ production)	Infections, autoinflammatory phenotype
Autosomal recessive CGD p47phox	*NCF1*	AR	608,512	N + M	Killing (faulty O_2_^−^ production)	Infections, autoinflammatory phenotype
Autosomal recessive CGD p67phox	*NCF2*	AR	608515	N + M	Killing (faulty O_2_^−^ production)	Infections, autoinflammatory phenotype
Autosomal recessive CGD p40phox	*NCF4*	AR	601488	N + M	Killing (faulty O_2_^−^ production)	Infections, autoinflammatory phenotype
G6PD deficiency class I	*G6PD*	XL	305900	N	Reduced O_2_^−^ production	Infections
4. Other non-lymphoid defects						
GATA2 deficiency (MonoMac syndrome)	*GATA2: loss of stem cells*	AD	137295	Monocytes + peripheral DC	Multi lineage cytopenias	Susceptibility to mycobacteria, HPV, histoplasmosis, alveolar proteinosis, MDS/AML/CMMoL, lymphedema
Congenital pulmonary alveolar proteinosis due to CSF2RB mutations	*CSF2RB*	AR	138981	Alveolar macrophages	GM-CSF signaling	Alveolar proteinosis
Congenital pulmonary alveolar proteinosis due to CSF2RA mutations	*CSF2RA*	XL (pseudoautosomal)	306250	Alveolar macrophages	GM-CSF signaling	Alveolar proteinosis

Total number of disorders in Table 5: 39. New disorders: 9, *WDR1*, *CFTR*, *SMARCD2*, *JAGN1*, *HYOU1*, *MKL1*, *DNAJC21*, *G6PD*, *CSF2RB*. Removed: cyclic neutropenia was merged with elastase deficiency

*MDS* myelodysplastic syndrome, *IUGR* intrauterine growth retardation, *LAD* leukocyte adhesion deficiency, *AML* acute myelogenous leukemia, *CMML* chronic myelomonocytic leukemia, *N* neutrophil, *M* monocyte, *MEL* melanocyte, *L* lymphocyte, *NK* natural killer, *XL* X-linked inheritance, *AR* autosomal recessive inheritance, *AD* autosomal dominant inheritance, *GOF* gain-of-function

**Table 6:** Defects in intrinsic and innate immunity

Disease	Genetic defect	Inheritance	OMIM	Affected cells	Affected function	Associated features
1. Mendelian susceptibility to mycobacterial disease (MSMD)						
IL-12 and IL-23 receptor β1 chain deficiency	*IL12RB1*	AR	601604	L + NK	IFN-γ secretion	Susceptibility to mycobacteria and *Salmonella*
IL-12p40 (IL-12 and IL-23) deficiency	*IL12B*	AR	161561	M	IFN-γ secretion	Susceptibility to mycobacteria and *Salmonella*
IFN-γ receptor 1 deficiency	*IFNGR1*	AR/AD	107470	M + L	IFN-γ binding and signaling	Susceptibility to mycobacteria and *Salmonella*
IFN-γ receptor 2 deficiency	*IFNGR2*	AR	147569	M + L	IFN-γ signaling	Susceptibility to mycobacteria and *Salmonella*
STAT1 deficiency (AD LOF)	*STAT1*	AD	600555	M + L	IFN-γsignaling	Susceptibility to mycobacteria, *Salmonella*
Macrophage gp91 phox deficiency	*CYBB*	XL	300481	Macrophage only	Killing (faulty O_2_^−^ production)	Isolated susceptibility to mycobacteria
IRF8 deficiency (AD)	*IRF8*	AD	601565	CD1c+ MDC	Differentiation of CD1c+ MDC subgroup	Susceptibility to mycobacteria
IRF8 deficiency (AR)	*IRF8*	AR	601565	CD1c+ MDC	Differentiation of CD1c+ MDC subgroup	Susceptibility to mycobacteria and multiple other infectious agents
Tyk2 deficiency	*TYK2*	AR	176941	Normal, but multiple cytokine signaling defect	Normal	Susceptibility to intracellular bacteria (mycobacteria, Salmonella), viruses, +/− elevated IgE
ISG15 deficiency	*ISG15*	AR	147571		IFNγ production defect	Susceptibility to mycobacteria (BCG), brain calcification
RORc deficiency	*RORC*	AR	602943	L + NK	Lack of functional RORγT protein, IFNγ production defect, complete absence of IL-17A/F-producing T cells	Susceptibility to mycobacteria and candida
JAK1 (LOF)	*JAK1*	AR	147795	N + L	IFNγ production	Susceptibility to mycobacteria and viruses, urothelial carcinoma
2. Epidermodysplasia verruciformis (HPV)						
EVER1 deficiency	*TMC6*	AR	605828	Keratinocytes and leukocytes	EVER proteins may be involved in the regulation of cellular zinc homeostasis in lymphocytes	Human papillomavirus (HPV) (group B1) infections and cancer of the skin (typical EV)
EVER2 deficiency	*TMC8*	AR	605829	Keratinocytes and leukocytes	EVER proteins may be involved in the regulation of cellular zinc homeostasis in Ly	HPV (group B1) infections and cancer of the skin (typical EV)
WHIM (warts, hypogammaglobulinemia, infections, myelokathexis) syndrome	*CXCR4*	AD GOF	162643	Granulocytes + lymphocytes	Increased response of the CXCR4 chemokine receptor to its ligand CXCL12 (SDF-1)	Warts, neutropenia, low B cell number, hypogammaglobulinemia
3. Predisposition to severe viral infection						
STAT1 deficiency (AR LOF)	*STAT1*	AR	600555	T and NK cells and monocytes	STAT1-dependent IFN-α, β, and γ response	Severe viral infections, mycobacterial infection
STAT2 deficiency	*STAT2*	AR	600556	T and NK cells	STAT2-dependent IFN-α, β, and γ response	Severe viral infections (disseminated vaccine-strain measles)
IRF7 deficiency	*IRF7*	AR	605047	Leukocytes, plasmacytoid dendritic cells, non-hematopoietic cells	IFN-α, β, and γ production and IFN-λ production	Severe influenza disease
IFNAR2 deficiency	*IFNAR2*	AR	602376	Broadly expressed	No response to IFN-α	Severe viral infections (disseminated vaccine-strain measles, HHV6)
CD16 deficiency	*FCGR3A*	AR	146740	NK cells	Altered NK cells function	Severe herpes viral infections, particularly VZV, Epstein-Barr virus (EBV), and (HPV)
MDA5 deficiency (LOF)	*IFIH1*	AR	606951	Somatic and hematopoietic	Viral recognition	Rhinovirus and other RNA viruses
4. Herpes simplex encephalitis (HSE)						
TLR3 deficiency	*TLR3*	AD or AR	603029	Central nervous system (CNS) resident cells and fibroblasts	TLR3-dependent IFN-α, β, and γ response	Herpes simplex virus 1 encephalitis (incomplete clinical penetrance for all etiologies listed here)
UNC93B1 deficiency	*UNC93B1*	AR	608204	CNS resident cells and fibroblasts	UNC-93B-dependent IFN-α, β, and γ response	Herpes simplex virus 1 encephalitis
TRAF3 deficiency	*TRAF3*	AD	601896	CNS resident cells and fibroblasts	TRAF3-dependent IFN-α, β, and γ response	Herpes simplex virus 1 encephalitis
TRIF deficiency	*TICAM1*	AD or AR	607601	CNS resident cells and fibroblasts	TRIF-dependent IFN-α, β, and γ response	Herpes simplex virus 1 encephalitis
TBK1 deficiency	*TBK1*	AD	604834	CNS resident cells and fibroblasts	TBK1-dependent IFN-α, β, and γ response	Herpes simplex virus 1 encephalitis
IRF3 deficiency	*IRF3*	AD	616532	CNS resident cells and fibroblasts	Low IFN-α/β production in response to HSV1 and decreased IRF3 phosphorylation	Herpes simplex virus 1 encephalitis
5. Predisposition to invasive fungal diseases						
CARD9 deficiency	*CARD9*	AR	607212	Mononuclear phagocytes	CARD9 signaling pathway	Invasive candidiasis infection, deep dermatophytoses, other invasive fungal infections
6. Predisposition to mucocutaneous candidiasis						
IL-17RA deficiency	*IL17RA*	AR	605461	Epithelial cells, fibroblasts, mononuclear phagocytes	IL-17RA signaling pathway	CMC, folliculitis
IL-17RC deficiency	*IL17RC*	AR	610925	Epithelial cells, fibroblasts, mononuclear phagocytes	IL-17RC signaling pathway	CMC
IL-17F deficiency	*IL17F*	AD	606496	T cells	IL-17F-containing dimers	CMC, folliculitis
STAT1 GOF	*STAT1*	AD GOF	600555	T cells, B cells, monocytes	Gain-of-function STAT1 mutations that impair the development of IL-17-producing T cells	CMC, various fungal, bacterial and viral (HSV) infections, autoimmunity (thyroiditis, diabetes, cytopenias), enteropathy
ACT1 deficiency	*TRAF3IP2*	AR	607043	T cells, fibroblasts	Fibroblasts fail to respond to IL-17A and IL-17F, and their T cells to IL-17E	CMC, blepharitis, folliculitis and macroglossia
7. TLR signaling pathway deficiency with bacterial susceptibility						
IRAK-4 deficiency	*IRAK4*	AR	606883	Lymphocytes + granulocytes + monocytes	TIR-IRAK4 signaling pathway	Bacterial infections (pyogens)
MyD88 deficiency	*MYD88*	AR	602170	Lymphocytes + granulocytes + monocytes	TIR-MyD88 signaling pathway	Bacterial infections (pyogens)
IRAK1 deficiency	*IRAK1*	XL	Not yet attributed	Lymphocytes + granulocytes + monocytes	TIR-IRAK1 signaling pathway	Bacterial infections, X-linked MECP2 deficiency-related syndrome due to a large de novo Xq28 chromosomal deletion encompassing both *MECP2* and *IRAK1*
TIRAP deficiency	*TIRAP*	AR	614382	Lymphocytes + granulocytes+ monocytes	TIRAP- signaling pathway, TLR1/2, TLR2/6, and TLR4 agonists were impaired in the fibroblasts and leukocytes	Staphylococcal disease during childhood
8. Other inborn errors of immunity related to non-hematopoietic tissues						
Isolated congenital asplenia (ICA) due to RPSA deficiency	*RPSA*	AD	271400	No spleen	RPSA encodes ribosomal protein SA, a component of the small subunit of the ribosome	Bacteremia (encapsulated bacteria)
Isolated congenital asplenia (ICA) due to HMOX deficiency	*HMOX*	AR	141250	Macrophages	HO-1 regulates iron recycling and heme-dependent damage occurs	Hemolysis, nephritis, inflammation
Trypanosomiasis	*APOL1*	AD	603743	Somatic	Lipid	Trypanosomiasis
Acute liver failure due to NBAS deficiency	*NBAS*	AR	608025	Somatic and hematopoietic	ER stress	Fever induces liver failure
Acute necrotizing encephalopathy	*RANBP2*	AD	601181	Ubiquitous expression	Nuclear pore	Fever induces acute encephalopathy
CLCN7 deficiency associated osteopetrosis	*CLCN7*	AR	602727	Osteoclasts	Secretory lysosomes	Osteopetrosis with hypocalcemia, neurologic features
SNX10 deficiency associated osteopetrosis	*SNX10*	AR	614780	Osteoclasts	Secretory lysosomes	Osteopetrosis with visual impairment
OSTM1 deficiency associated osteopetrosis	*OSTM1*	AR	607649	Osteoclasts	Secretory lysosomes	Osteopetrosis with hypocalcemia, neurologic features
PLEKHM1 deficiency associated osteopetrosis	*PLEKHM1*	AR	611466	Osteoclasts	Secretory lysosomes	Osteopetrosis
TCIRG1 deficiency associated osteopetrosis	*TCIRG1*	AR	604592	Osteoclasts	Secretory lysosomes	Osteopetrosis with hypocalcemia
TNFRSF11A deficiency associated osteopetrosis	*TNFRSF11A*	AR	603499	Osteoclasts	Osteoclastogenesis	Osteopetrosis
TNFSF11 deficiency associated osteopetrosis	*TNFSF11*	AR	602642	Stromal	Osteoclastogenesis	Osteopetrosis with severe growth retardation
NCSTN deficiency hidradenitis suppurativa	*NCSTN*	AD	605254	Epidermis	Gamma-secretase in hair follicle regulates keratinization	Hidradenitis suppurativa with acne
PSEN deficiency hidradenitis suppurativa	*PSEN*	AD	104311	Epidermis	Gamma-secretase in hair follicle regulates keratinization	Hidradenitis suppurative with cutaneous hyperpigmentation
PSENEN deficiency hidradenitis suppurativa	*PSENEN*	AD	607632	Epidermis	Gamma-secretase in hair follicle regulates keratinization	Hidradenitis suppurativa

Total number of disorders in Table 6: 52. New genes: 19, *IFNAR* ***2***, *IRF3*, *JAK1*, *IRAK1*, *TIRAP*, *IFIH1*, *HMOX*, *NBAS*, *RANBP2*, *CLCN7*, *SNX10*, *OSTM1*, *PLEKHM1*, *TCIRG1*, *TNFRSF11A*, *TNFSF11*, *NCSTN*, *PSEN*, *PSENEN*

*NF-κB* nuclear factor kappa B, *TIR* Toll and interleukin-1 receptor, *IFN* interferon, *TLR* Toll-like receptor, *MDC* myeloid dendritic cell, *CNS* central nervous system, *CMC* chronic mucocutaneous candidiasis, *HPV* human papillomavirus, *VZV* varicella zoster virus, *EBV* Epstein-Barr virus, *HHV6* human herpesvirus 6, *XL* X-linked inheritance, *AR* autosomal recessive inheritance, *AD* autosomal dominant inheritance, *LOF* loss-of-function, *GOF* gain-of-function

**Table 7:** Autoinflammatory disorders

1. Type 1 interferonopathies							
Disease	Genetic defect	Inheritance	OMIM	T cells	B cells	Functional defect	Associated features
TREX1 deficiency, Aicardi-Goutieres syndrome 1 (AGS1)	*TREX1*	AR or AD	606609	Not assessed	Not assessed	Intracellular accumulation of abnormal ss DNA species leading to increased type I IFN production	Classical AGS, SLE, FCL
RNASEH2B deficiency, AGS2	*RNASEH2B*	AR	610326	Not assessed	Not assessed	Intracellular accumulation of abnormal RNA-DNA hybrid species leading to increased type I IFN production	Classical AGS, SP
RNASEH2C deficiency, AGS3	*RNASEH2C*	AR	610330	Not assessed	Not assessed	Intracellular accumulation of abnormal RNA-DNA hybrid species leading to increased type I IFN production	Classical AGS
RNASEH2A deficiency, AGS4	*RNASEH2A*	AR	606034	Not assessed	Not assessed	Intracellular accumulation of abnormal RNA-DNA hybrid species leading to increased type I IFN production	Classical AGS
SAMHD1 deficiency, AGS5	*SAMHD1*	AR	606754	Not assessed	Not assessed	Controls dNTPs in the cytosol, failure of which leads to increased type I IFN production	Classical AGS, FCL
ADAR1 deficiency, AGS6	*ADAR1*	AR	146920	Not assessed	Not assessed	Catalyzes the deamination of adenosine to inosine in dsRNA substrates, failure of which leads to increased type I IFN production	Classical AGS, BSN, SP
Aicardi-Goutieres syndrome 7 (AGS7)	*IFIH1* (GOF)	AD	606951	Not assessed	Not assessed	IFIH1 gene encodes a cytoplasmic viral RNA receptor that activates type I interferon signaling through the MAVS adaptor molecule	Classical AGS, SLE, SP, SMS
Spondyloenchondro-dysplasia with immune dysregulation (SPENCD)	*ACP5*	AR	171640	Not assessed	Not assessed	Upregulation of IFN through mechanism possibly relating to pDCS	Short stature, SP, ICC, SLE, thrombocytopenia and autoimmune hemolytic anemia, possibly recurrent bacterial and viral infections
STING-associated vasculopathy, infantile-onset	*TMEM173*	AR	612374	Not assessed	Not assessed	STING activates both the NF-kappa-B and IRF3 transcription pathways to induce expression of IFN	Skin vasculopathy, inflammatory lung disease, systemic autoinflammation and ICC, FCL
X-linked reticulate pigmentary disorder	*POLA1*	XL	301220	Not assessed	Not assessed	POLA1 is required for synthesis of cytosolic RNA:DNA and its deficiency leads to increase production of type I interferon	Hyperpigmentation, characteristic facies, lung and GI involvement
USP18 deficiency	*USP18*	AR	607057	Not assessed	Not assessed	Defective negative regulation of ISG15 leading to increased IFN	TORCH like syndrome
CANDLE (chronic atypical neutrophilic dermatitis with lipodystrophy)	*PSMB8* ^a^	AR and AD	256040	Not assessed	Not assessed	Mutations cause increased IFN signaling through an undefined mechanism	Contractures, panniculitis, ICC, fevers
Singleton-Merten syndrome	*DDX58*	AD	609631	Not assessed	Not assessed	Recognizes double stranded RNA	Dental dysplasia), calcifications in the aorta, osteoporosis, especially in the hands and feet
2. Defects affecting the inflammasome							
Disease	Genetic defect	Inheritance	OMIM	Affected cells	Functional defects	Associated features	
Familial Mediterranean fever	MEFV	AR or AD	249100 134610	Mature granulocytes, cytokine-activated monocytes	Decreased production of pyrin permits ASC-induced IL-1 processing and inflammation following subclinical serosal injury, macrophage apoptosis decreased	Recurrent fever, serositis and inflammation responsive to colchicine. Predisposes to vasculitis and inflammatory bowel disease	
Mevalonate kinase deficiency (Hyper IgD syndrome)	MVK	AR	260920	Somatic and hemaotpoietic	Affecting cholesterol synthesis, pathogenesis of disease unclear	Periodic fever and leukocytosis with high IgD levels	
Muckle-Wells syndrome	NLRP3 (also called NALP3 CIAS1 or PYPAF1)	AD GOF	191900	PMNs Monocytes	Defect in cryopyrin, involved in leukocyte apoptosis and NFkB signaling and IL-1 processing	Urticaria, SNHL, amyloidosis	
Familial cold autoinflammatory syndrome 1	NLRP3	AD GOF	120100	PMNs, monocytes	As above	Non-pruritic urticaria, arthritis, chills, fever and leukocytosis after cold exposure	
Familial cold autoinflammatory syndrome 2	NLRP12	AD GOF	611762	PMNs, monocytes	As above	Non-pruritic urticaria, arthritis, chills, fever and leukocytosis after cold exposure	
Neonatal onset multisystem inflammatory disease (NOMID) or chronic infantile neurologic cutaneous and articular syndrome (CINCA)	NLRP3	AD GOF	607115	PMNs, chondrocytes	As above	Neonatal onset rash, chronic meningitis, and arthropathy with fever and inflammation	
NLRC4-MAS (macrophage activating syndrome) or familial cold autoinflammatory syndrome 4	NLRC4	AD GOF	616050 616115	PMNs monocytes macrophages	Gain-of-function mutation in NLRC4 results in elevated secretion of IL-1β and IL-18 as well as macrophage activation	Severe enterocolitis and macrophage activation syndrome	
PLAID (PLCγ2 associated antibody deficiency and immune dysregulation) or familial cold autoinflammatory syndrome 3 or APLAID (c2120A>C)	*PLCG2*	AD GOF	614468	B cells, NK, mast cells	Mutations cause activation of IL-1 pathways	Cold urticaria hypogammaglobulinemia, autoinflammation	
NLRP1 deficiency	NLRP1	AR	606579	Leukocytes	Systemic elevation of IL-18 and caspase 1, suggesting involvement of NLRP1 inflammasome	Dyskeratosis, autoimmunity and arthritis	
3. Non-inflammasome-related conditions							
Disease	Genetic defect	Inheritance	OMIM	Affected cells	Functional defects	Associated Features	
TNF receptor-associated periodic syndrome (TRAPS)	*TNFRSF1A*	AD	142680	PMNs, monocytes	Mutations of 55-kD TNF receptor leading to intracellular receptor retention or diminished soluble cytokine receptor available to bind TNF	Recurrent fever, serositis, rash, and ocular or joint inflammation	
Pyogenic sterile arthritis, pyoderma gangrenosum, acne (PAPA) syndrome, hyperzincemia, and hypercalprotectinemia	*PSTPIP1* (also called *C2BP1*)	AD	604416	Hematopoietic tissues, upregulated in activated T cells	Disordered actin reorganization leading to compromised physiologic signaling during inflammatory response	Destructive arthritis, inflammatory skin rash, myositis	
Blau syndrome	*NOD2* (also called CARD15)	AD	186580	Monocytes	Mutations in nucleotide binding site of CARD15, possibly disrupting interactions with lipopolysaccharides and NF-kB signaling	Uveitis, granulomatous synovitis, camptodactyly, rash and cranial neuropathies, 30% develop Crohn colitis	
ADAM17 deficiency	*ADAM17*	AR	614328	Leukocytes and epithelial cells	Defective TNFα production	Early-onset diarrhea and skin lesions	
Chronic recurrent multifocal osteomyelitis and congenital dyserythropoietic anemia (Majeed syndrome)	*LPIN2*	AR	609628	Neutrophils, bone marrow cells	Undefined	Chronic recurrent multifocal osteomyelitis, transfusion-dependent anemia, cutaneous inflammatory disorders	
DIRA (deficiency of the interleukin-1 receptor antagonist)	*IL1RN*	AR	612852	PMNs, Monocytes	Mutations in the IL-1 receptor antagonist allow unopposed action of interleukin-1	Neonatal onset of sterile multifocal osteomyelitis, periostitis and pustulosis	
DITRA (deficiency of IL-36 receptor antagonist)	*IL36RN*	AR	614204	Keratinocytes, leukocytes	Mutations in IL-36RN leads to increase IL-8 production	Pustular psoriasis	
SLC29A3 mutation	*SLC29A3*	AR	602782	Leukocytes, bone cells		Hyperpigmentation hypertrichosis, histiocytosis-lymphadenopathy plus syndrome	
CAMPS (CARD14 mediated psoriasis)	*CARD14*	AD	602723	Mainly in keratinocytes	Mutations in CARD14 activate the NF-kB pathway and production of IL-8	Psoriasis	
Cherubism	*SH3BP2*	AD	118400	Stroma cells, bone cells	Hyperactived macrophage and increase NF-kB	Bone degeneration in jaws	
COPA defect	*COPA*	AD	6011924	PMN and tissue specific cells	Defective intracellular transport via the coat protein complex I (COPI)	Autoimmune inflammatory arthritis and interstitial lung disease with Th17 dysregulation and autoantibody production	
Otulipenia/ORAS	*OTULIN*	AR	615712	Leukocytes	Increase LUBAC induction of NF-KB activation leading to high proinflammatory cytokines levels	Fever, diarrhea, dermatitis	
A20 deficiency	*TNFAIP3*	AD LOF	616744	Lymphocytes	Defective inhibition of NF-KB signaling pathway	Arthralgia, mucosal ulcers, ocular inflammation	
ADA2 deficiency	*CECR1*	AR	607575	Lymphocytes	ADAs deactivate extracellular adenosine and terminate signaling through adenosine receptors	Polyarteritis nodosa, childhood-onset, early-onset recurrent ischemic stroke and fever	
AP1S3 deficiency	*AP1S3*	AR	615781	Keratinocytes	Disrupted TLR3 translocation	Pustular psoriasis	

Total number of disorders in Table 7: 37. New disorders: 7, *DDX58*, *POLA1*, *USP18*, *NLRP1*, *OTULIN*, *TNFAIP3*, *AP1S3*

*IFN* interferon; *HSM* hepatosplenomegaly; *CSF* cerebrospinal fluid; *SLE* systemic lupus erythematosus; *TORCH* toxoplasmosis, other, rubella, cytomegalovirus, and herpes infections; *SNHL* sensorineural hearing loss; *AGS* Aicardi-Goutières syndrome; *BSN* bilateral striatal necrosis; *FCL* familial chilblain lupus; *ICC* intracranial calcification; *IFN* interferon type I; *pDCs* plasmacytoid dendritic cells; *SP* spastic paraparesis; *SMS* Singleton-Merten syndrome; *ss* single-stranded DNA; *XL* X-linked inheritance; *AR* autosomal recessive inheritance; *AD* autosomal dominant inheritance; *LOF* loss-of-function; *GOF* gain-of-function

^a^Variants in *PSMB4*, *PSMB9*, *PSMA3*, and *POMP* have been proposed to cause a similar CANDLE phenotype in monogenic and digenic models

**Table 8:** Complement deficiencies

1. Complement deficiencies					
Disease	Genetic defect	Inheritance	Gene OMIM	Laboratory features	Associated features
C1q deficiency due to defects in C1QA	*C1QA*	AR	120550	Absent CH50 hemolytic activity, defective activation of the classical pathway, diminished clearance of apoptotic cells	SLE, infections with encapsulated organisms
C1q deficiency due to defects in C1QB	*C1QB*	AR	120570	Absent CH50 hemolytic activity, Defective activation of the classical pathway, diminished clearance of apoptotic cells	SLE, infections with encapsulated organisms
C1q deficiency due to defects in C1QC	*C1QC*	AR	120575	Absent CH50 hemolytic activity, Defective activation of the classical pathway, diminished clearance of apoptotic cells	SLE, infections with encapsulated organisms
C1r deficiency	*C1R*	AR	613785	Absent CH50 hemolytic activity, defective activation of the classical pathway	SLE, infections with encapsulated organisms, Ehlers-Danlos phenotype
C1s deficiency	*C1S*	AR	120580	Absent CH50 hemolytic activity, defective activation of the classical pathway	SLE, infections with encapsulated organisms, Ehlers-Danlos phenotype
Complete C4 deficiency	*C4A + C4B*	AR	120810	Absent CH50 hemolytic activity, defective activation of the classical pathway, complete deficiency requires biallelic mutations/deletions/conversions of both C4A and C4B	SLE, infections with encapsulated organisms, partial deficiency is common (either C4A or C4B) and appears to have a modest effect on host defense
C2 deficiency	*C2*	AR	217000	Absent CH50 hemolytic activity, defective activation of the classical pathway	SLE, infections with encapsulated organisms, atherosclerosis
C3 deficiency (LOF)	*C3*	AR	120700	Absent CH50 and AH50 hemolytic activity, defective opsonization, defective humoral immune response	Infections, glomerulonephritis, atypical hemolytic-uremic syndrome with GOF mutations
C3 GOF	*C3*	AD	120700	Increased activation of complement	Atypical hemolytic-uremic syndrome
C5 deficiency	*C5*	AR	120900	Absent CH50 and AH50 hemolytic activity Defective bactericidal activity	Disseminated neisserial infections
C6 deficiency	*C6*	AR	217050	Absent CH50 and AH50 hemolytic activity, defective bactericidal activity	Disseminated neisserial infections
C7 deficiency	*C7*	AR	217070	Absent CH50 and AH50 hemolytic activity, defective bactericidal activity	Disseminated neisserial infections
C8α deficiency	*C8A*	AR	120950	Absent CH50 and AH50 hemolytic activity, defective bactericidal activity	Disseminated neisserial infections
C8γ deficiency	*C8G*	AR	120930	Absent CH50 and AH50 hemolytic activity, defective bactericidal activity	Disseminated neisserial infections
C8β-deficiency	*C8B:*	AR	120960	Absent CH50 and AH50 hemolytic activity, defective bactericidal activity	Disseminated neisserial infections
C9 deficiency	*C9*	AR	120940	Reduced CH50 and AP50 hemolytic activity, deficient bactericidal activity	Mild susceptibility to disseminated neisserial infections
MASP2 deficiency	*MASP2*	AR	605102	Deficient activation of the lectin activation pathway	Pyogenic infections, inflammatory lung disease, autoimmunity
Ficolin 3 deficiency	*FCN3*	AR	604973	Absence of complement activation by the Ficolin 3 pathway.	Respiratory infections, abscesses
C1 inhibitor deficiency	*SERPING1*	AD	606860	Spontaneous activation of the complement pathway with consumption of C4/C2, spontaneous activation of the contact system with generation of bradykinin from high molecular weight kininogen	Hereditary angioedema
Factor B GOF	*CFB*	AD	138470	Gain-of-function mutation with increased spontaneous AH50	Atypical hemolytic-uremic syndrome
Factor B LOF	*CFB*	AR	138470	Deficient activation of the alternative pathway	Infections with encapsulated organisms
Factor D deficiency	*CFD*	AR	134350	Absent AH50 hemolytic activity	Neisserial infections
Properdin deficiency	*CFP*	XL	300383	Absent AH50 hemolytic activity	Neisserial infections
Factor I deficiency	*CFI*	AR	217030	Spontaneous activation of the alternative complement pathway with consumption of C3	Infections, disseminated neisserial infections, atypical hemolytic-uremic syndrome, preeclampsia
Factor H deficiency	*CFH*	AR or AD	134370	Spontaneous activation of the alternative complement pathway with consumption of C3	Infections, disseminated neisserial infections, atypical hemolytic-uremic syndrome, preeclampsia
Factor H-related protein deficiencies	*CFHR1-5*	AR or AD	134371, 600889, 605336, 605337, 608593	Normal CH50, AH50, autoantibodies to factor H, linked deletions of one or more CFHR genes leads to susceptibility autoantibody-mediated aHUS	Older onset atypical hemolytic-uremic syndrome, disseminated neisserial infections
Thrombomodulin deficiency	*THBD*	AD	188040	Normal CH50, AH50	Atypical hemolytic-uremic syndrome
Membrane cofactor protein (CD46) deficiency	*CD46*	AD	120920	Inhibitor of complement alternate pathway, decreased C3b binding	Atypical hemolytic-uremic syndrome, infections, preeclampsia
Membrane attack Complex inhibitor (CD59) deficiency	*CD59*	AR	107271	Erythrocytes highly susceptible to complement-mediated lysis	Hemolytic anemia, polyneuropathy
CD55 deficiency (CHAPEL disease)	*CD55*	AR	125240	Hyperactivation of complement on endothelium	Protein losing enteropathy, thrombosis

Total number of disorders in Table 8: 30. New disorders: 1, *CD55*

*MAC* membrane attack complex, *SLE* systemic lupus erythematosus, *XL* X-linked inheritance, *AR* autosomal recessive inheritance, *AD* autosomal dominant inheritance, *LOF* loss-of-function, *GOF* gain-of-function

**Table 9:** Phenocopies of inborn errors of immunity

1. Phenocopies of inborn errors of immunity					
Disease	Genetic defect/presumed pathogenesis	Circulating T cells	Circulating B cells	Serum Ig	Associated features/similar PID
Associated with somatic mutations					
Autoimmune lymphoproliferative syndrome (ALPS–SFAS)	Somatic mutation in *TNFRSF6*	Increased CD4−CD8− double negative (DN) T alpha/beta cells	Normal, but increased number of CD5+ B cells	Normal or increased	Splenomegaly, lymphadenopathy, autoimmune cytopenias, defective lymphocyte apoptosis/ALPS–FAS (=ALPS type Im)
RAS-associated autoimmune leukoproliferative disease (RALD)	Somatic mutation in *KRAS* (GOF)	Normal	B cell lymphocytosis	Normal or increased	Splenomegaly, lymphadenopathy, autoimmune cytopenias, granulocytosis, monocytosis/ALPS-like
RAS-associated autoimmune leukoproliferative disease (RALD)	Somatic mutation in *NRAS* (GOF)	Increased CD4−CD8− double negative (DN) T alpha/beta cells	Lymphocytosis	Normal or increased	Splenomegaly, lymphadenopathy, autoantibodies/ALPS-like
Cryopyrinopathy, (Muckle-Wells/CINCA/NOMID-like syndrome)	Somatic mutation in *NLRP3*	Normal	Normal	Normal	Urticaria-like rash, arthropathy, neurological signs
Hypereosinophilic syndrome due to somatic mutations in STAT5b	Somatic mutation in STAT5b (GOF)	Normal	Normal	Normal	Eosinophilia, atopic dermatitis, urticarial rash, diarrhea
Large granular lymphocytosis	Somatic mutations in STAT3 (GOF)	Clonal expansion of large T cells	Normal	Normal	Anemia, neutropenia, splenomegaly
Associated with autoantibodies					
Chronic mucocutaneous candidiasis (isolated or with APECED syndrome)	Germline mutation in *AIRE* AutoAb to IL-17 and/or IL-22	Normal	Normal	Normal	Endocrinopathy, chronic mucocutaneous candidiasis/CMC
Adult-onset immunodeficiency with susceptibility to mycobacteria	AutoAb to IFNγ	Decreased naive T cells	Normal	Normal	Mycobacterial, fungal, *Salmonella* VZV infections/MSMD, or CID
Recurrent skin infection	AutoAb to IL-6	Normal	Normal	Normal	Staphylococcal infections/STAT3 deficiency
Pulmonary alveolar proteinosis	AutoAb to GM-CSF	Normal	Normal	Normal	Pulmonary alveolar proteinosis, cryptococcal meningitis, disseminated nocardiosis/CSF2RA deficiency
Acquired angioedema	AutoAb to CI inhibitor	Normal	Normal	Normal	Angioedema/*C1 INH* deficiency (hereditary angioedema)
Atypical hemolytic-uremic syndrome	AutoAb to complement factor H	Normal	Normal	Normal	aHUS = spontaneous activation of the alternative complement pathway
Thymoma with hypogammaglobulinemia (Good syndrome)	AutoAb to various cytokines	Increased CD8+ T cells	No B cells	Decreased	Invasive bacterial, viral or opportunistic infections, autoimmunity, PRCA, lichen planus, cytopenia, colitis, chronic diarrhea

Total number of conditions for Table 9: 12

*aHUS* atypical hemolytic-uremic syndrome, *GOF* gain-of-function, *PRCA* pure red cell aplasia

The goal of the IUIS Expert Committee on Primary Immunodeficiencies is to increase awareness, facilitate recognition, promote optimal treatment, and support research in the field of immune deficiency disorders. Thus, the “IUIS PID Committee Report on Inborn Errors of Immunity” and “Update of the Phenotypical IUIS Classification for Primary Immunodeficiencies” publications are important resources for clinicians and researchers. In addition, these tables form the basis of lists used for sequencing panels and are used to monitor health utilization which will influence health services funding by federal or state governments and/or insurance companies in various global settings. The addition of ICD10 codes for the online version will promote a harmonization between the diagnostic tables and coding items that will facilitate bioinformatics research going forward.
